# BeMADS1 is a key to delivery MADSs into nucleus in reproductive tissues-*De novo* characterization of *Bambusa edulis* transcriptome and study of MADS genes in bamboo floral development

**DOI:** 10.1186/1471-2229-14-179

**Published:** 2014-07-02

**Authors:** Ming-Che Shih, Ming-Lun Chou, Jin-Jun Yue, Cheng-Tran Hsu, Wan-Jung Chang, Swee-Suak Ko, De-Chih Liao, Yao-Ting Huang, Jeremy JW Chen, Jin-Ling Yuan, Xiao-Ping Gu, Choun-Sea Lin

**Affiliations:** 1 Agricultural Biotechnology Research Center, Academia Sinica, Taipei, Taiwan; 2 Department of Life Sciences, Tzu Chi University, Hualien, Taiwan; 3 Research Institute of Subtropical Forestry, Chinese Academy of Forestry, Fuyang, China; 4 Biotechnology Center in Southern Taiwan, Academia Sinica, Tainan, Taiwan; 5 Department of Computer Science and Information Engineering, National Chung Cheng University, Chia-yi, Taiwan; 6 Institute of Biomedical Sciences, National Chung-Hsing University, Taichung, Taiwan

**Keywords:** Hybrid transcriptomics, Protein translocation, *In vitro* flowering, ABCDE model, *In situ* hybridization, Juvenility

## Abstract

**Background:**

The bamboo *Bambusa edulis* has a long juvenile phase *in situ*, but can be induced to flower during *in vitro* tissue culture, providing a readily available source of material for studies on reproductive biology and flowering. In this report, *in vitro*-derived reproductive and vegetative materials of *B. edulis* were harvested and used to generate transcriptome databases by use of two sequencing platforms: Illumina and 454. Combination of the two datasets resulted in high transcriptome quality and increased length of the sequence reads. In plants, many MADS genes control flower development, and the ABCDE model has been developed to explain how the genes function together to create the different whorls within a flower.

**Results:**

As a case study, published floral development-related OsMADS proteins from rice were used to search the *B. edulis* transcriptome datasets, identifying 16 *B. edulis* MADS (*BeMADS*). The *BeMADS* gene expression levels were determined qRT-PCR and *in situ* hybridization. Most *BeMADS* genes were highly expressed in flowers, with the exception of *BeMADS34*. The expression patterns of these genes were most similar to the rice homologs, except *BeMADS18* and *BeMADS34*, and were highly similar to the floral development ABCDE model in rice. Transient expression of MADS-GFP proteins showed that only BeMADS1 entered leaf nucleus. BeMADS18, BeMADS4, and BeMADS1 were located in the lemma nucleus. When co-transformed with BeMADS1, BeMADS15, 16, 13, 21, 6, and 7 translocated to nucleus in lemmas, indicating that BeMADS1 is a key factor for subcellular localization of other BeMADS.

**Conclusion:**

Our study provides abundant *B. edulis* transcriptome data and offers comprehensive sequence resources. The results, molecular materials and overall strategy reported here can be used for future gene identification and for further reproductive studies in the economically important crop of bamboo.

## Background

Bamboo is important not only to human industry, but also in the environment and for animal habitat. Because bamboo has a long juvenile phase, an unpredictable flowering time and dies after flowering, it is difficult to investigate its reproductive biology. In large bamboo forests, bamboo flowering can cause economic and ecological damage. For example, in 1970–80, a widespread flowering of the bamboos *Bashania fangiana* and *Fargesia denudata* in China threatened the food source of pandas in the affected area [1]. In 1963–73, two-thirds of the *Phyllostachys bambusoides* stands were flowering in Japan, limiting the bamboo industry [2]. Therefore, the mechanism timing bamboo flowering is of interest outside academic pursuits. To investigate this topic, a reliable source of reproductive materials is required. Using tissue culture, bamboo can be induced to flower [3] by addition of cytokinin [4]. Additionally, vegetative shoots can be induced by auxin treatment [5]. Using tissue culture, genomic resources have been established for the bamboo *Bambusa edulis*, including microarray [6] and Expressed Sequence Tag (EST) libraries [7].

Next Generation Sequencing (NGS) has been employed to supplement the microarray and EST libraries for non-model plants [8,9]. This method was also applied to the bamboos *Dendrocalamus latiflorus*[10,11] and *P. heterocycla*[12]. The *Bambusa* genus comprises more than one hundred species, which are widely distributed in the tropical and subtropical areas of Asia, Africa, and Oceania. There are many important economic species, such as *B. edulis* and *B. oldhamii*, which are grown for human consumption, *B. pervariabilis* and *B. tuldoides*, which are grown for building and furniture supplies, *B. textilis* and *B. rigida*, which are grown for fiber, and *B. ventricosa* and *B. multiplex var. riviereorum*, which are grown for ornamental use. Additionally, *Bambusa* has been used for cross-breeding with other bamboo genera [13]. Compared with the transcriptome resources of *Dendrocalamus* and *Phyllostachys*, the transcriptome data from *Bambusa* is limited.

Generally, flower morphology is diverse and unique, and therefore serves as an excellent material for taxonomic and evolutionary studies [14]. Recent studies on floral development-related genes in dicot plants can be understood by the ABCDE model of flower initiation [15,16]. A and B class genes cooperate to form the petal. B and C class genes cooperate to form the stamen. A whorl that only expresses a C class gene develops into a carpel. D class genes are related to ovule identity. E class genes are expressed in all four whorls of floral organs and ovule and correlate to the floral meristem determinacy [16-18]. Interestingly, all genes thus far identified in this model, except *AP2*, which belongs to the APETALA2/ ethylene-responsive element binding protein (AP2/EREBP) family, are MADS genes. MADS genes encode transcription factors. Based on amino acid sequences, these genes can be divided into two types: type I (SRF-like) and type II (MEF-like). In plants, the MEF-like MADS-domain proteins contain four conserved domains: the MADS (M) domain, the Intervening (I) domain, the Keratin-like (K) domain and a C-terminal domain. Therefore, these type II proteins are called MIKC-type MADS-box proteins. All MADS genes in the ABCDE model of plant floral development are MIKC-type MADS.

The ABCDE model was developed through research in dicot plants. However, the monocots, specifically the family Poaceae, contain important cereal crops, such as rice (*Oryza sativa*), maize (*Zea mays*), wheat (*Triticum* spp.), and barley (*Hordeum vulgare*) [19]. Together with bamboo, these species form the Bambusoideae, Ehrhartoideae (rice) and Pooideae (Wheat, barley and oats; BEP) phylogenetic clade. Similarities and differences in the genetic sequences and expression patterns of floral development genes in this clade are informative for both macroevolution [20] and agricultural application. Furthermore, since monocot flower development can directly affect the grain yield, the mechanism of flowering is an important topic in Poaceae research. Additionally, the morphology of monocot floral organs is different from that in the dicots. In rice and bamboo, the inflorescence is composed of spikelets. Each spikelet contains one floret. The floret is divided into four whorls, namely: lemma and palea (whorl 1), two lodicules (whorl 2), six stamens (whorl 3), and gynoecium (one ovary and two stigmas, whorl 4) [21]. In rice, MADS genes have been identified and divided into the ABCDE gene classes [20-28]. Compared with rice (*Oryza sativa*), relatively fewer MADS-box genes have been characterized in bamboo [29-31]. Therefore, to systematically study MADS-box genes involved in floral formation in bamboo, the *B. edulis* NGS transcriptome databases were developed and searched to identify putative flower development-related MADS (*BeMADS*) genes.

## Results and discussion

### RNA-Seq, de novo assembly and sequence analysis

Three *B. edulis* transcriptome libraries (454, Illumina and Hybrid, Additional file 1) were constructed from RNA derived from different developmental stages and various tissues *in vitro* (roots, stems, leaves and flowers). To comprehensively cover the *B. edulis* transcriptome, equal amounts of total RNA from each sample were pooled together before the mRNA was isolated, enriched, sheared into smaller fragments, and reverse-transcribed into cDNA. We performed RNA-Seq analyses by either Roche 454 or Illumina sequencing platforms based on the two-phase assembly approach. The resulting sequencing data were subjected to bioinformatic analysis.

**Figure 1:**
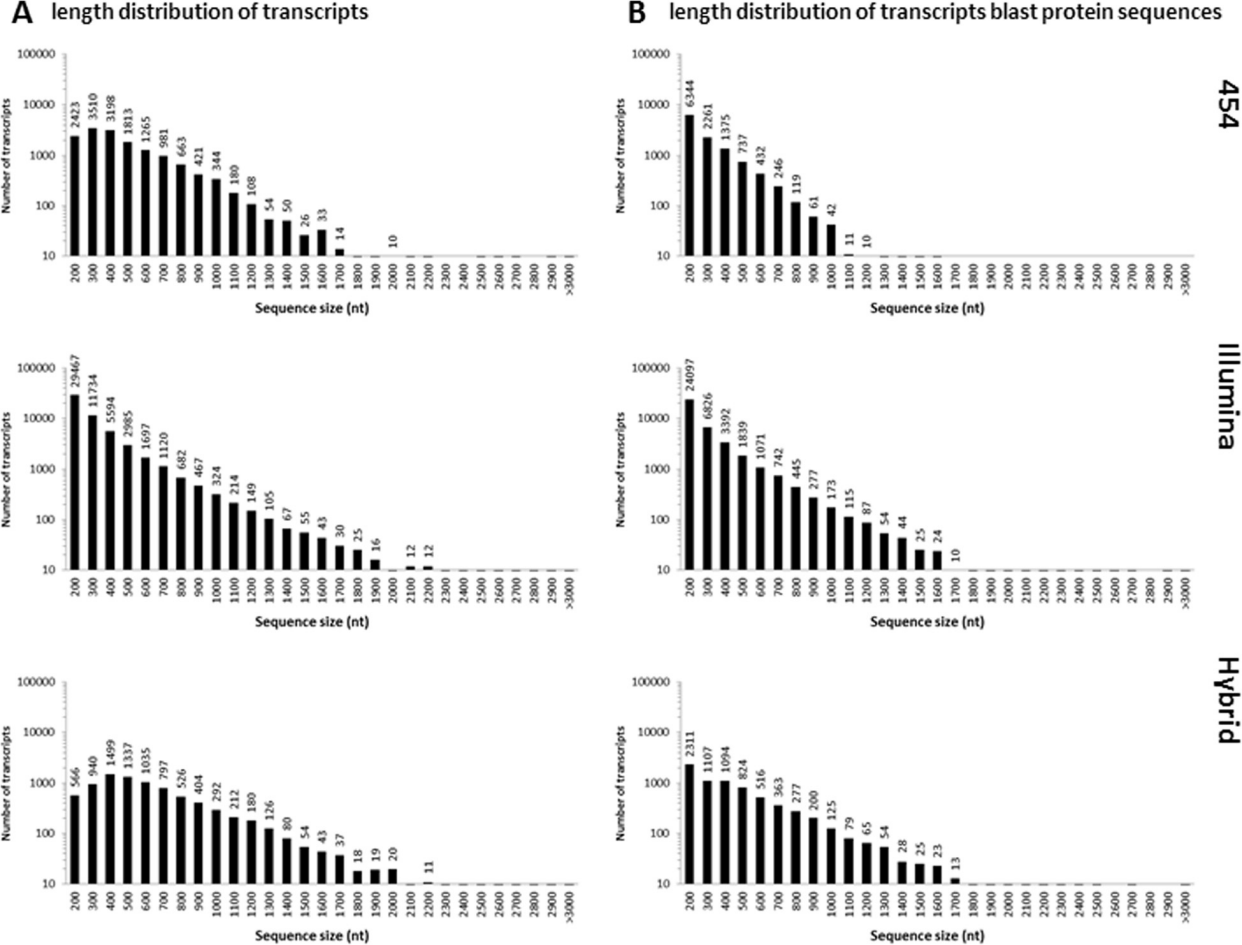
**Overview of sequence reads and assembly of the three *B. edulis *transcriptomes.** The length distribution of the contigs obtained from *de novo* assembly of high-quality, clean reads from NGS data across three datasets, namely sequence data from Roche 454, Illumina, and Hybrid transcriptome. **Panel A** shows the lengths of all contigs from each dataset. **Panel B**: shows the contig lengths for only those contigs that had BLASTX hits in the NCBI protein database.

The size distribution of the *B. edulis* unigenes identified from the three transcriptome datasets is shown in Figure 1A and Table 1. These set of unigenes were annotated using BLASTX searches of a variety of protein databases, taking into account the identity between the unigene sequence and the sequence in the database (E-value ≤10^-5^). The size distributions of the BLAST-aligned predicted proteins in the three *B. edulis* transcriptome datasets are shown in Figure 1B.

**Table 1:** **Sequence assembly results from three *B. edulis *** **transcriptome databases**

	**Platform**	**Unigene**	**Total length (nt)**	**Min length (bp)**	**Max length (bp)**	**Mean length (bp)**	**N50**	**GC percentage**	**N percentage**
454	454	15,117	7,824,977	200	4,347	518	562^a^	46.84%	0.01%
Illumina	Illumina	54,830	19,681,401	200	4,666	359	361^b^	50.44%	0.23%
Hybrid	454+Illumina	8,241	5,517,588	200	4,666	670	730^c^	48.02%	0.13%

^a^50% of the assembled bases were incorporated into contigs of 562 bp or longer.

^b^50% of the assembled bases were incorporated into unigenes of 361 bp or longer.

^c^50% of the assembled bases were incorporated into unigenes of 730 bp or longer.

Currently, there are several NGS platforms, i.e. Illumina/Solexa Genome Analyzer, Roche 454 GS FLX and Applied Biosystems SOLiD, used in genome and transcriptome research, each with advantages and weaknesses. In research using NGS, the accuracy and length of the sequences are important. For instance, while the read length obtained using the Sanger method is longer, the method is more expensive. Illumina technology has higher sequencing coverage, resulting in higher accuracy, but the read length is short, making it difficult to obtain long contigs during *de novo* assembly. Therefore, integration of multiple sequencing platforms is one strategy for *de novo* sequencing when there is no reference genome available [32]. Through a hybrid assembly, contigs averaging 670 nts were constructed, an average length longer than that reported for the *D. latiflorus* transcriptomes, which only used Illumina methods [10,11].

Some pre-assembled sequences were lost during the integration of the Illumina and 454 sequences. Therefore, in this report, the transcriptomes derived from each sequencing platform are also presented. This allowed searches for DNA sequences of interest in two *de novo* transcriptomes and one virtual hybrid transcriptome, with the results further assembled after hunting in the three databases.

### Functional annotation of *B. edulis* transcriptome

**Figure 2:**
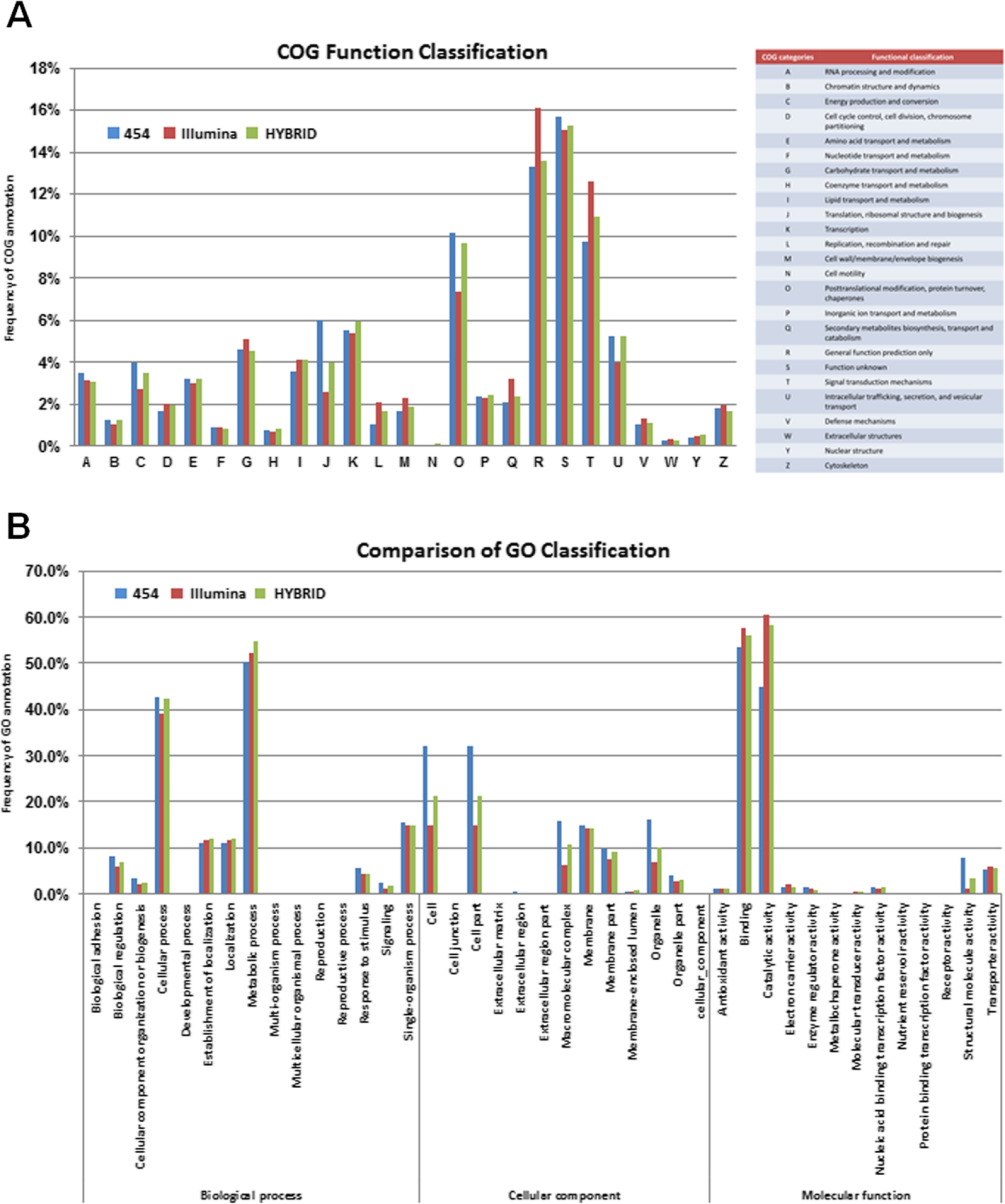
**Assignment of COG and GO classifications to *B. edulis *unigenes across three transcriptome datasets. A**. COG functional classification of the *B. edulis* transcriptome. The graph shows the percentage of the whole dataset that was annotated within any one COG function.A total of 9,347 (for 454 dataset), 29,654 (for Illumina dataset) and 6,158 (for Hybrid dataset) unigenes showed significant homologies to genes in the COG protein database and were distributed into 25 COG categories (A-Z, except X). **B**. GO classification of the *B. edulis* transcriptome. The graph shows the percentage of the whole dataset that was annotated within any one GO sub-category. A total of 15,916 unigenes from 454 dataset were distributed into 36 GO sub-categories (functional groups), 38,740 unigenes from Illumina dataset were distributed into 41 sub-categories, and 10,866 unigenes from Hybrid dataset were distributed into 34 sub-categories.

To predict the function of these assembled transcripts, non-redundant sequences were submitted to a BLASTX (E-value ≤ 10^-5^) search against the following databases: Gene Ontology (GO), NCBI non-redundant database (Nr), Swiss-Prot, Kyoto Encyclopedia of Genes and Genomes pathway (KEGG) and Orthologous Groups of proteins (COG) (Additional files 2 and 3). Nearly 77.0% (11,646 unigenes for 454 dataset), 71.6% (39,261 unigenes for Illumina dataset) and 86.7% (7,141 unigenes for Hybrid dataset) of all predicted unigenes significantly matched a sequence in at least one of the four databases used for annotation (Additional files 2, 3, and Figure 2). In order to determine if a complete representation of the known genes within a gene family could be found in our datasets, the MADS gene family was used for further transcriptome validation.

### Sixteen putative BeMADS genes identified from *B. edulis* transcriptome database

Using16 floral-specific rice MADS protein sequences, 16 *BeMADS* genes were identified (Table 2, accession no. is shown in Additional file 4). When using the data from only one sequencing platform, most of the sequences were partial and some could not be identified. For example, *BeMADS2, 5, 8, 14, 15,* and *18* were not found in the Illumina database. *BeMADS4, 7, 13, 21,* and *34* were not found in the 454 database. Combining the sequences from the three databases resulted in identification of full-length transcripts for *BeMADS1, 2, 3, 4, 8, 14, 15, 16* and *58* (Table 2). These results indicated that combination of different sequencing platforms resulted in longer sequence lengths and more complete transcriptome assembly. The same observation was made in the *Phalaenopsis* transcriptome study [32].

**Table 2:** **The 16 BeMADS genes - similar to rice floral development-related MADS - were identified from *B. edulis *** **transcriptomes and *B. oldhamii *** **BAC library**

**Gene**	**Illumina dataset**	**454 dataset**	**Hybrid dataset**	**Orthologous rice gene**	**Protein identity**
*BeMADS1*	Unigene49607	isogroup06012, isogroup03511	Bamboo_rep_c34	*OsMADS1*	77.2% (244/257)
*BeMADS2*	-	isogroup00569	Bamboo_rep_c1172	*OsMADS2*	92.3% (209/209)
*BeMADS3*	Unigen16863	isogroup02737	Bamboo_c1430, Bamboo_c5032	*OsMADS3*	88.3% (236/287)
*BeMADS4*	Unigene31768	-	Bamboo_c4955	*OsMADS4*	83.3% (209/210)
*BeMADS5*^*#*^	-	isogroup07515	Bamboo_c4877	*OsMADS5*	86.4% (228/225)
*BeMADS6*^*#*^	Unigene274	isogroup00332	Bamboo_c2324	*OsMADS6*	89.7% (272/250)
*BeMADS7*^*#*^	Unigene48557; Unigene26633	-	Bamboo_c7627	*OsMADS7*	83.5% (246/310)
*BeMADS8*	-	isogroup00335	Bamboo_rep_c1395	*OsMADS8*	88.8% (247/248)
*BeMADS13*^*#*^	Unigene50193	-	-	*OsMADS13*	83.3% (249/270)
*BeMADS14*	-	isogroup03309	Bamboo_rep_c2518	*OsMADS14*	91.9% (244/253)
*BeMADS15*	-	isogroup00461	-	*OsMADS15*	86.2% (261/267)
*BeMADS16*	Unigene27646	isogroup00922	Bamboo_c5509	*OsMADS16*	90.4% (230/224)
*BeMADS18*^*#*^	-	isogroup01124	-	*OsMADS18*	77.6% (255/249)
*BeMADS21*^*#*^	Unigene39623	-	-	*OsMADS21*	78.7% (252/265)
*BeMADS34*^*#*^	-	-	-	*OsMADS34*	81.1% (218/239)
*BeMADS58*	Unigene53551	isogroup02355	-	*OsMADS58*	85.2% (230/233)

-: no homologous sequence was identified in this database.

#: full length genes were identified by BAC sequences.

The high sequence homology in the MADS gene family, especially the highly conserved M domain in the N-terminal region, can be a problem in distinguishing between paralogs during *de novo* assembly and promoter walking. To identify the promoter region and to clone full length genes, a BAC strategy was used [8,33-35] to identify 7 additional full length *BeMADS* genes in *B. oldhamii* (Table 2).

In addition to a sequencing strategy, it is possible to search databases from other closely related species. According to chloroplast genome results, *P. heterocycla, D. latiflorus* and *B. oldhamii* are highly homologous species [36,37]. Some bamboo gene sequences, including genomic, full-length cDNA, and EST, have been published [7,10-12]. From the NCBI database, *P. heterocycla* and *D. latiflorus* MADS genes were identified. Integration of the data from different bamboo species will prove important not only for gene identification, but also for evolutionary studies.

### Evolutionary relationships among bamboo and other monocot MADS genes similar to genes in the ABCDE model of floral development

**Figure 3:**
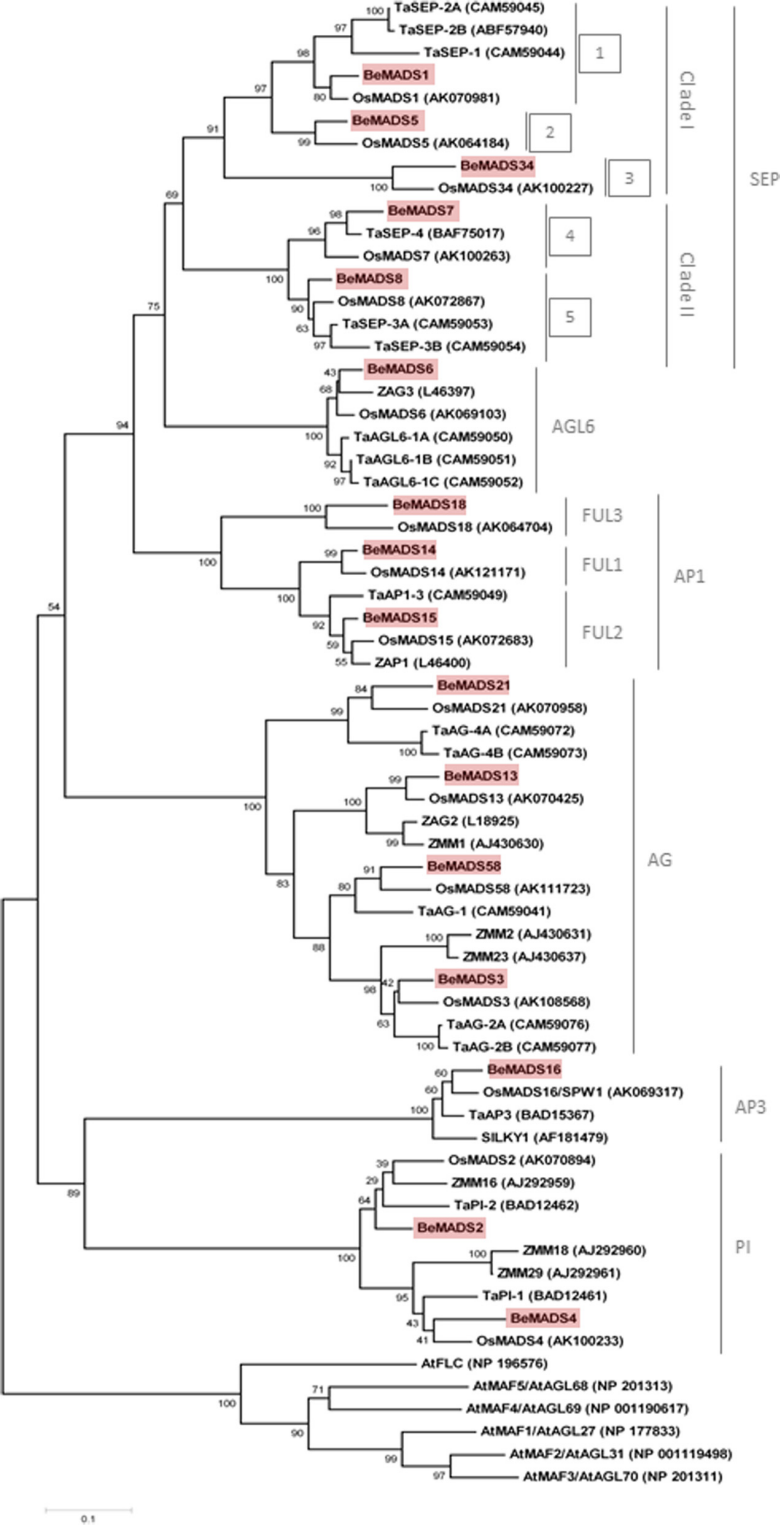
**Phylogenetic tree based on amino acid sequences of MIKC-type MADS-box genes.** 60 MIKC-type MADS-box genes were used: 16 from *Bambusa edulis*, 16 from rice (*Oryza sativa*), 10 from maize (*Zea mays*), and 18 from wheat (*Triticum aestivum* L*.*). Deduced full-length amino acid sequences were used for the alignments. The phylogenetic tree was constructed by the neighbor-joining method and evaluated by bootstrap analysis (MEGA version 4.0). Numbers on major branches indicate bootstrap percentage for 1,000 replicates. Six Arabidopsis sequences of the FLC subfamily were used as outgroups. Proteins from *B. edulis* were highlighted with *red boxes*. The three grass clades of FUL1, FUL2, and FUL3 within the AP1 subfamily and the two major clades of the SEP subfamily are labeled on the *right*. The five grass clades within the SEP subfamily are indicated by *numbers* showing their respective name according to previous studies [41], namely 1: LHS1/OsMADS1, 2: OsMADS5, 3: OsMADS34, 4: OsMADS7/45, 5: OsMADS8/24. Subfamilies of the plant MIKC-type genes and the functional classification according to the A/B/C/D/E classes are indicated at the *right margin*.

To identify the putative functional classification of the bamboo BeMADS in relation to the ABCDE model and to understand the phylogenetic relationships with other known MADS-box genes regulating floral development, we collected full-length amino acid sequences of MADS from bamboo (16), rice (16) [38], maize (10) [39] and wheat (18) [40] to perform phylogenetic analysis (Figure 3). Our phylogenetic tree is organized with an overlay of the ABCDE model classes for ease of discussion, based on this [28].

BeMADS14, BeMADS15 and BeMADS18 belonged to the AP1 family in the A class (Figure 3), which includes the FUL1, FUL2 and FUL3 clades [20,41]. BeMADS14, like OsMADS14, belonged to the FUL1 clade. BeMADS15 sorted into the FUL2 clade, close to ZAP1 from maize and OsMADS15 from rice. BeMADS18, like OsMADS18, belonged to FUL3 clade. These genes, identified as transcripts from *B. edulis*, clustered with genes that were hypothesized to occur twice in grass genomes due to duplication events [20].

BeMADS2, BeMADS4 and BeMADS16 were most orthologous to the B class proteins (Figure 3). BeMADS2 and BeMADS4 belong to the PI family, with BMADS2 closely related to OsMADS2 and maize ZMM2, and BeMADS4 most closely related to OsMADS4 (Figure 3). BeMADS16 was most closely related to OsMADS16 (SPW1) in the AP3 clade. The presence of one AP3 ortholog (BeMADS16) and two PI orthologs (BeMADS2, BeMADS4) is similar to the other monocots and bolsters the hypothesis that early in the evolution of the monocots there was an ancient gene duplication event of the PI ortholog [21,42].

Four proteins, BeMADS3, BeMADS13, BeMADS21 and BeMADS58, belong to the AG family (Figure 3), which functionally classifies as a C/D class MADS protein. In the C class functional group, BeMADS3 and BeMADS58 were most closely related to rice OsMADS3 and OsMADS58, respectively. BeMADS13 and BeMADS21 were most orthologous to the D class functional group and closely related to rice OsMADS13 and OsMADS21, respectively (Figure 3). Based on the phylogenetic tree analysis, the D class had four subclades in the grasses, and each subclade contained at least one gene from rice, maize or wheat. The AG family of proteins is divided between the C and D classes, the first of which contains the rice proteins OsMADS3 and OsMADS58 – which are like AG, SHATTERPROOF1 (SHP1), and SHATTERPROOF2 (SHP2) in Arabidopsis - and the second of which contains OsMADS13 and OsMADS21 – which are like SEEDSTIK (STK) in Arabidopsis [43]. Our data show that the bamboo BeMADS proteins in the C/D group also contain one gene in each subclade (Figure 3), which can be interpreted as a major gene duplication event that occurred in both grass C and D clades before the separation of the maize, rice, wheat and bamboo lineages [44,45].

Five proteins, *BeMADS1*, *BeMADS5*, *BeMADS7*, *BeMADS8*, and *BeMADS34*, were most closely related to the SEP family, which belongs to the E functional group (Figure 3). The class E genes in rice belong to two clades - the *SEP*-clade (Clade II) and the *LOFSEP*-clade (Clade I) [41]. The *OsMADS1/LEAFY HULL STERILE 1* (*LHS1*), *OsMADS5/OSM5*, and *OsMADS34/PANICLE PHYTOMER 2* (*PAP2*) grouped into the *LOFSEP*-clade [46]. While this class can be divided into multiple layers of derived clades, the most informative may be the five distinct subclades, 1–5 [40,41]. This phylogenetic division indicates that the bamboo BeMADS genes in E-group are closely related to the OsMADSs in each clade and that at least one BeMADS falls into each subclade (Figure 3), similar to the homologous genes identified from rice, maize and wheat. According to these results, the common ancestor of these species may contain at least five *SEP*-like genes.

There is one family of MIKC-MADS that does not have a defined role in the ABCDE model, the AGL6 clade. Recently, it was reported that *OsMADS6/MOSAIC FLORAL ORGANS 1* (*MFO1*) plays a synergistic role in regulating floral organ identity, floral meristem determinacy and meristem fate with class B (*OsMADS16*), C (*OsMADS3*), and D (*OsMADS13*) genes and with the *YABBY* gene *DROOPING LEAF* (*DL*), which was previously known to function in carpel specification [28,47,48]. These results suggest that rice *AGL6*-clade gene may have an E-class function. Our phylogenetic analysis indicates that BeMADS6 belongs to the AGL6 family and is most similar to OsMADS6 (Figure 3). Past phylogenetic analysis showed that *AGL6*-like genes are sister to the *SEP*-like genes [49]. Interestingly, *SEP* genes were only identified in angiosperms, but *AGL6*-like genes were identified in both angiosperms and gymnosperms.

As a whole, this phylogenetic tree shows that bamboo contains MADS proteins not only in each putative functional group but also in each sub-clade and that the BeMADS are most often sister to the rice OsMADS. Therefore, functional experiments in bamboo can be designed based on previous work in monocots. Recently, two *AP1*/*SQUA-*like MADS-box genes from bamboo (*Phyllostachys praecox*), *PpMADS1* (FUL3 subfamily) and *PpMADS2* (FUL1 subfamily), were found to play roles in floral transition, since they caused early flowering through upregulation of *AP1* when overexpressed in Arabidopsis. Yeast two-hybrid experiments demonstrated that PpMADS1 and PpMADS2 might interact with different partners to play a part in floral transition of bamboo [31].

### *BeMADS* gene expression

**Figure 4:**
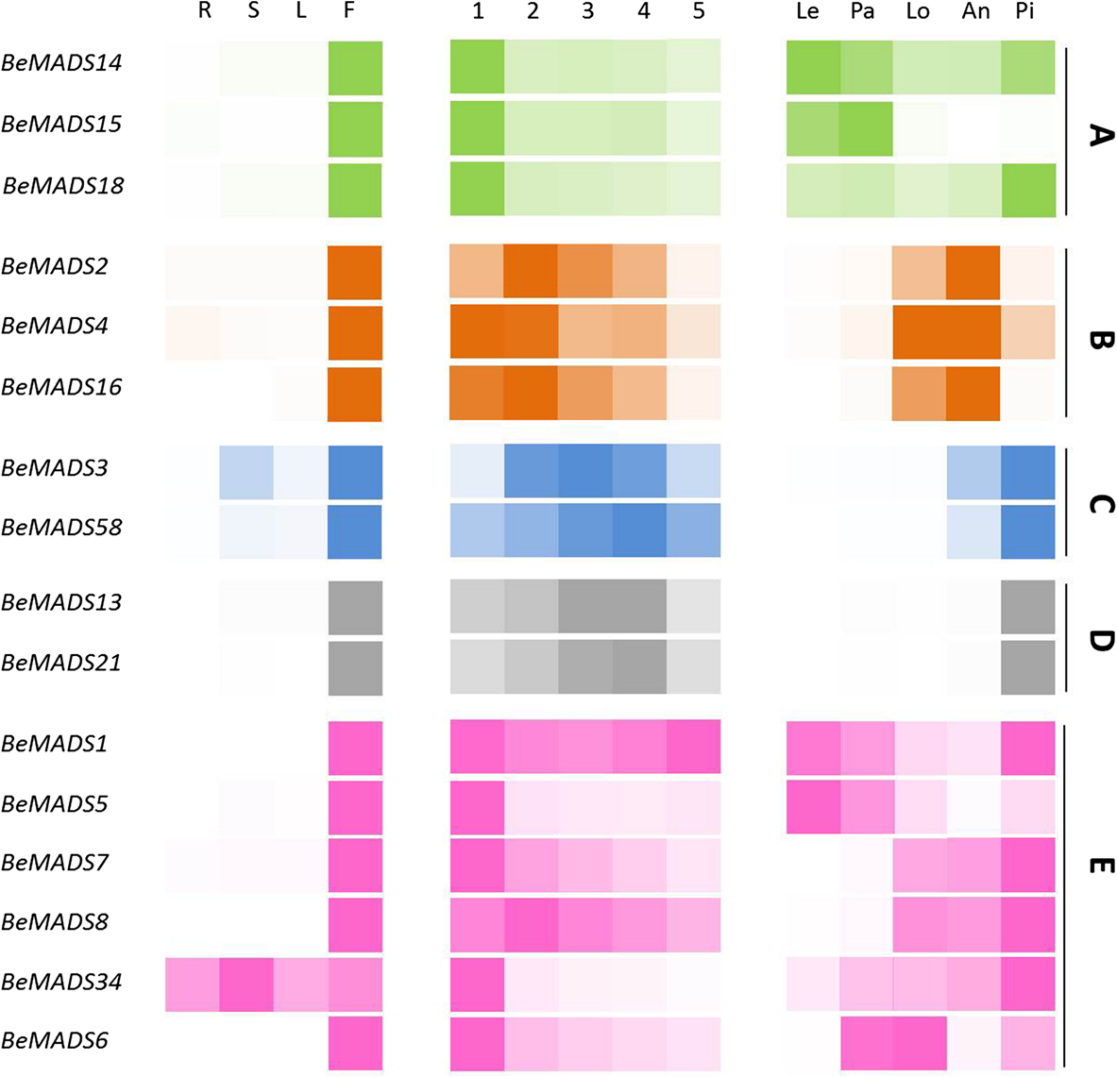
**Developmental stage, organ and tissue-specific expression patterns of *BeMADS *genes.** B. *edulis* RNA was extracted from different *in vitro* tissues and subjected to cDNA synthesis: R: roots; L: leaves; S: stems; F: flowers; 1–5: young to old florets, see Additional file 5; and the floral organs Le: lemma; Pa: palea; Lo: lodicules; An: anther; and Pi: pistil. Quantitative RT-PCR was undertaken using the primers in Additional file 6. The *B. edulis* tubulin gene was used as the internal control. The color intensity is related to the expression level, with darker indicating higher expression. The colors represent the classes of the gene from Figure 3: **A**: green, **B**: orange, **C**: blue, **D**: grey, **E**: pink.

The expression patterns of the 16 *BeMADS* were analyzed by real-time quantitative RT-PCR using gene-specific primer sets across several tissue types and floret ages (Figure 4). Data are grouped by functional classes, A-E, on the right. Most of the *BeMADS* genes were highly expressed in the floral organ (F). *BeMADS34* was expressed in various tissues, but most highly expressed in stem (S). This result is different to the presumed ortholog in rice, *OsMADS34*, which is ubiquitously expressed but highly expressed in spikelet and has been shown to be involved in inflorescence and spikelet development [50].

The process of bamboo flower development can be divided into 5 stages, from small floral buds to mature flower (stages 1–5). The expression level of the A-, B- and E-class *BeMADS* genes were high in the youngest floral buds (stage 1) and decreased through floral maturity. The expression of C- and D-class *BeMADS* genes were reduced in stage 1, slightly increased in stages 3 to 4, and decreased in stage 5 (Figure 4). Expression of *BeMADS* in class E showed two overall patterns, one that was high throughout floral development and one that was high just in stage 1.

We further analyzed the expression patterns of the *BeMADSs* in bamboo floral organs. From the outer whorl to the inner whorl within the floral organ, we divided the flower into lemma (Le, whorl 1), palea (Pa, whorl 1), lodicule (Lo, whorl 2), anther (An, whorl 3) and pistil (Pi, whorl 4). Our results showed that for the A- class genes, *BeMADS14* was expressed throughout, but higher in the lemma and pistil, *BeMADS15* was expressed in the lemma and palea, and *BeMADS18* was most highly expressed in the pistil (Figure 4). The *BeMADS14* homolog *OsMADS14* was only detected in inflorescence and developing caryopses by transcript analysis [51]. Based on *in situ* hybridization analysis, *OsMADS14* was expressed in the early spikelet meristem, the primordia of flower organs, and the reproductive organs, but did not express in the vegetative organs [51]. These data are consistent with that of *BeMADS14*, which was only expressed in floral organ (Figure 4). The *BeMADS15* homolog *OsMADS15* was first detected in the spikelet meristem and then in vegetative organs only after emergence of spikelet organs, including lodicules, palea, lemma, and glumes [52]. *BeMADS15* showed a similar expression pattern, but very low expression in the lodicules (Figure 4), same like the ortholog in wheat, *TaAP1-3*[40]. The expression pattern of *BeMADS18* was different from the rice ortholog *OsMADS18* and the wheat ortholog *TaAP1-2. OsMADS18* is expressed in roots, leaves, inflorescences, and developing kernels, but not in young seedlings. The *OsMADS18* transcript was also detected in leaves following germination after four weeks and increased during the reproductive phase [22]. A similar gene expression pattern was also found for wheat *TaAP1-2*, which is highly expressed in roots, stems, leaves, different developmental stages of spikes and different spikelet organs, including the glumes, lemma, and palea [40]. It is interesting that *TaAP1-2* was also expressed at low levels in developing caryopses, lodicules, stamens and pistils [40]. However, our result showed that *BeMADS18* was more highly expressed in the fourth whorl (pistil) than in other whorls in the floral organ. While *BeMADS18* is classified into the A class by sequence similarity and phylogenetic analysis, its expression pattern differs somewhat from typical A-class genes from other grasses. Perhaps *BeMADS18* functions in pistil formation with other functional genes in the C or E class.

**Figure 5:**
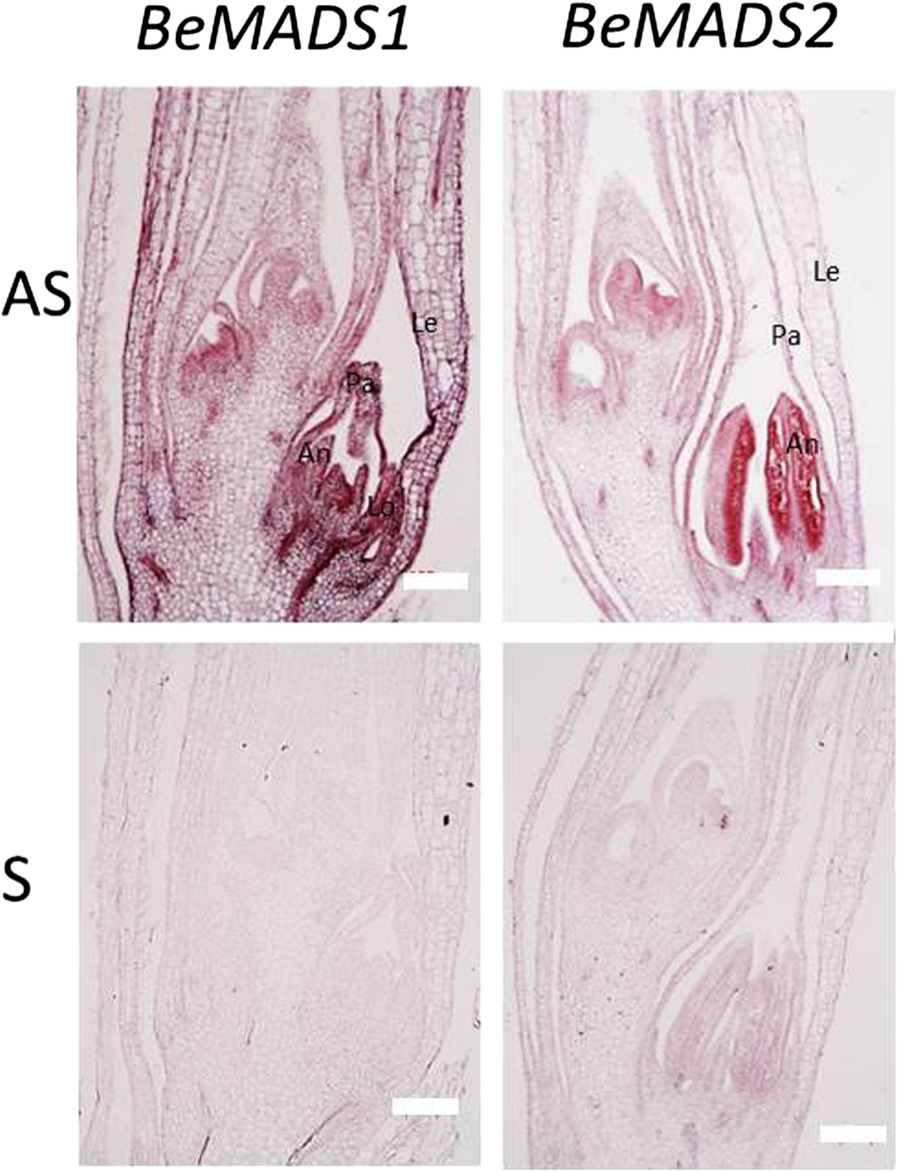
**In situ localization of *BeMADS1 *and *BeMADS2 *transcripts in early floral bud of *B. edulis*.** Longitudinal sections were hybridized with DIG-labeled antisense and sense probes. Left: Hybridization signals of antisense (upper) and sense (lower) probe of *BeMADS1*. Right: Hybridization signals of antisense (upper) and sense (lower) probe of *BeMADS2*. The signals detected from sense probe were used as negative control. Pa: palea; Lo: lodicules; An: anther. Bar = 100 μm

A single copy of an *AP3/DEF*-like gene but two copies of the *PI/GLO*-like genes is a phenomenon common in other plant species, including Arabidopsis, *Antirrhinum*, rice, maize, and wheat, and also bamboo (Figure 3). B class genes are required to specify petal and stamen identity [53]. Whether of *PI/GLO* or *AP3/DEF* lineage, the mRNA of B class genes (*BeMADS2*, *BeMADS4* and *BeMADS16*) showed a similar expression pattern: mainly in flower, with low levels detected in lemma and palea, but high levels in lodicules and anthers (Figure 4). This may indicate redundant function as a safety measure to insure flower development. Transcripts of the *AP3/DEF*-like *OsMADS16*/*SPW1* and maize *SILKY1* (*SL*) were detected mainly in the lodicules and stamen primordia during floral development, but not in developing carpels [21,24]. The expression patterns of *BeMADS16* and wheat *TaAP3* are similarly in mature female organs [40], but the function of *TaAP3* is unknown. The *PI/GLO*-like *BeMADS2* and *BeMADS4* display similar expression patterns, but *BeMADS2* was highly expressed in anthers and *BeMADS4* was highly expressed in lodicules. However, *BeMADS2* and *BeMADS4* expression patterns were still similar to other members of the *PI* family in the floral organ [40,42,52]. Rice *in situ* hybridization data showed that in the late stage of floral development *OsMADS2* mRNA was not detected in the glumes, lemma, palea, pistil primordia or developing pistils, but limited to and highly expressed in lodicules. Expression in stamens occurred in later developmental stages once all the floral organs were differentiated [52]. To further explore the spatial and temporal expression pattern of *BeMADS2* in early floral bud development of bamboo, we investigated the expression pattern of genes by *in situ* hybridization. *BeMADS2* was highly expressed in the anthers of second flowers (Figure 5). This result correlated with the qRT-PCR data (Figure 4). We also found that *BeMADS4* and *BeMADS2* showed similar expression patterns to wheat orthologs *TaPI*-*1* and *TaPI*-*2*, including the initial expression in spike primordia and later expression in developing caryopses (5 days after anthesis), lodicules, stamens, and pistils from fully emerged spikes [40].

The C class genes are part of the *AG*-lineage and include *BeMADS3* and *BeMADS58*, which were mainly expressed in the floral bud and then later in anthers and pistils, with especially high levels in pistils (Figure 4). This result is consistent with the involvement of the C class genes in development of the third (stamen) and fourth (carpel) whorls [26]. A similar result was also found for the other C class genes *OsMADS3*, *OsMADS58*, *TaAG*-*1*, and *TaAG*-*2*. In rice, *in situ* hybridization results indicated that *OsMADS3* and *OsMADS58* were limited to stamens, carpels, and ovule primordia. Only *OsMADS3* was strongly expressed in the presumptive region from which the stamen, carpel, and ovule primordia subsequently differentiate, whereas *OsMADS58* remained during differentiation and development [26]. Wheat *TaAG*-*1* and *TaAG*-*2* transcripts gradually increased during spike development and were only detected in the stamens and pistils [40]. The spatial and temporal expression of *BeMADS3* and *BeMADS58* requires further analysis.

The D class genes also belong to the *AG*-lineage and include *BeMADS13* and *BeMADS21*, which were mainly expressed in flower and concentrated in pistils (Figure 4). This expression pattern of D class genes was consistent with the gene function in ovule identity determination and floral meristem determinacy [44]. The D class genes *OsMADS13*, maize *ZAG2* and Arabidopsis *STK* have a similar expression pattern in floral organs [44,54,55]. The gene expression of rice *OsMADS21* was very low in developing anthers, carpels, styles/stigmas, and ovule [44]. During the late stage of flower development, *OsMADS21* was particularly evident in the inner cell layers of the ovary and in the ovule integuments, an expression region that overlapped with that of *OsMADS13*[44]. Based on the qRT-PCR results, the expression amount was no different between *BeMADS13* and *21*. The expression localization was also similar: highly expressed in pistil.

The E class genes, such as Arabidopsis *SEPALLATA* (*SEP*), function in specification of sepal, petal, stamen, carpel, and ovule [16,56] and interact with genes from the other four ABCD groups at the protein level to form higher order MADS-box protein complexes that control the development of the fourth whorls within the flower [16,17,56-58]. The E class genes in the *SEP* lineage in bamboo were *BeMADS1*, *BeMADS5*, *BeMADS7*, *BeMADS8* and *BeMADS34. BeMADS6* was located in the *AGL6* lineage. The six genes were expressed in various flower structures, but were most highly expressed in the lemma (*BeMADS1* and *BeMADS5*), lodicule (*BeMADS7* and *BeMADS8*), and pistil (*BeMADS1*, *BeMADS5*, *BeMADS7*, *BeMADS8* and *BeMADS34*) for the 5 *SEP*-like genes and in the palea and lodicule for the *AGL6*-like *BeMADS6* (Figure 4). The expression pattern of E class genes in rice differed from BeMADS in the same group, such as the *BeMADS1* homolog *OsMADS1. OsMADS1* was not detected before glume primordia emergence, after which it was mainly present in the spikelet meristem, and then limited to the lemma and palea, with very low expression in the carpel [59]. *BeMADS1* was expressed through the entire flower development, at all examined stages and tissues, but was highly expressed in the pistil, moderately expressed in lemma and anther, and very limited in anthers and lodicules (Figure 4). We also investigated the spatial and temporal expression pattern of *BeMADS1* in early floral bud development of bamboo by *in situ* hybridization. Our result showed that the transcripts of *BeMADS1* could also be detected in the pistil (Figure 5), correlating with the expression pattern determined by qRT-PCR (Figure 4). The other E class genes in rice, *OsMADS7* and *OsMADS8*, were first detected in spikelet meristems, were not in lemma or palea primordia at a later stage, but were found in developing lodicules, stamens, and carpels during spikelet development [27]. Our result also showed that *BeMADS7* and *BeMADS8* have similar expression patterns in floral organs, but low levels in the anthers (Figure 4). The expression of *BeMADS34* was high in the fourth whorl (pistils) (Figure 4) and differed to that of its rice ortholog *OsMADS34*, which was initially expressed throughout the floral meristem and subsequently detected in palea, lemma, and the sporogenic tissue of the anthers in the mature flower [51]. A previous expression study showed a grass *AGL6*-like gene to mainly express in the inflorescence [60]. The *BeMADS6* homolog in rice, *OsMADS6*, was first detected in the floral meristem and later in palea, lodicules, and pistil and at lower levels in stamens [48]. This similar expression pattern in floral organs was also shown for *BeMADS6* (Figure 4).

In summary, we used transcriptomics to identify 16 *BeMADS* genes and used amino acid homology to cluster them according to their similarity to genes in the ABCDE model of floral development. Gene expression analysis demonstrated, except for *BeMADS18* and *34*, that most *BeMADS* have similar expression patterns during flower development as their better studied orthologous genes in rice.

### Subcellular localization of BeMADS proteins

The putative functions of all the BeMADS proteins are as transcription factors. The localization of these proteins was predicted to be nuclear. To investigate the subcellular localization of BeMADS family members, *B. edulis* leaves and lemmas were used for transient transformation of GFP-BeMADS fusions (leaves: Additional file 7, lemmas: Figure 5). Except for some of the signal for BeMADS1-YFP, the fifteen BeMADS proteins, representing each of the 5 classes, were found throughout the cytoplasm when transiently expressed in leaves (Additional file 7).

**Figure 6:**
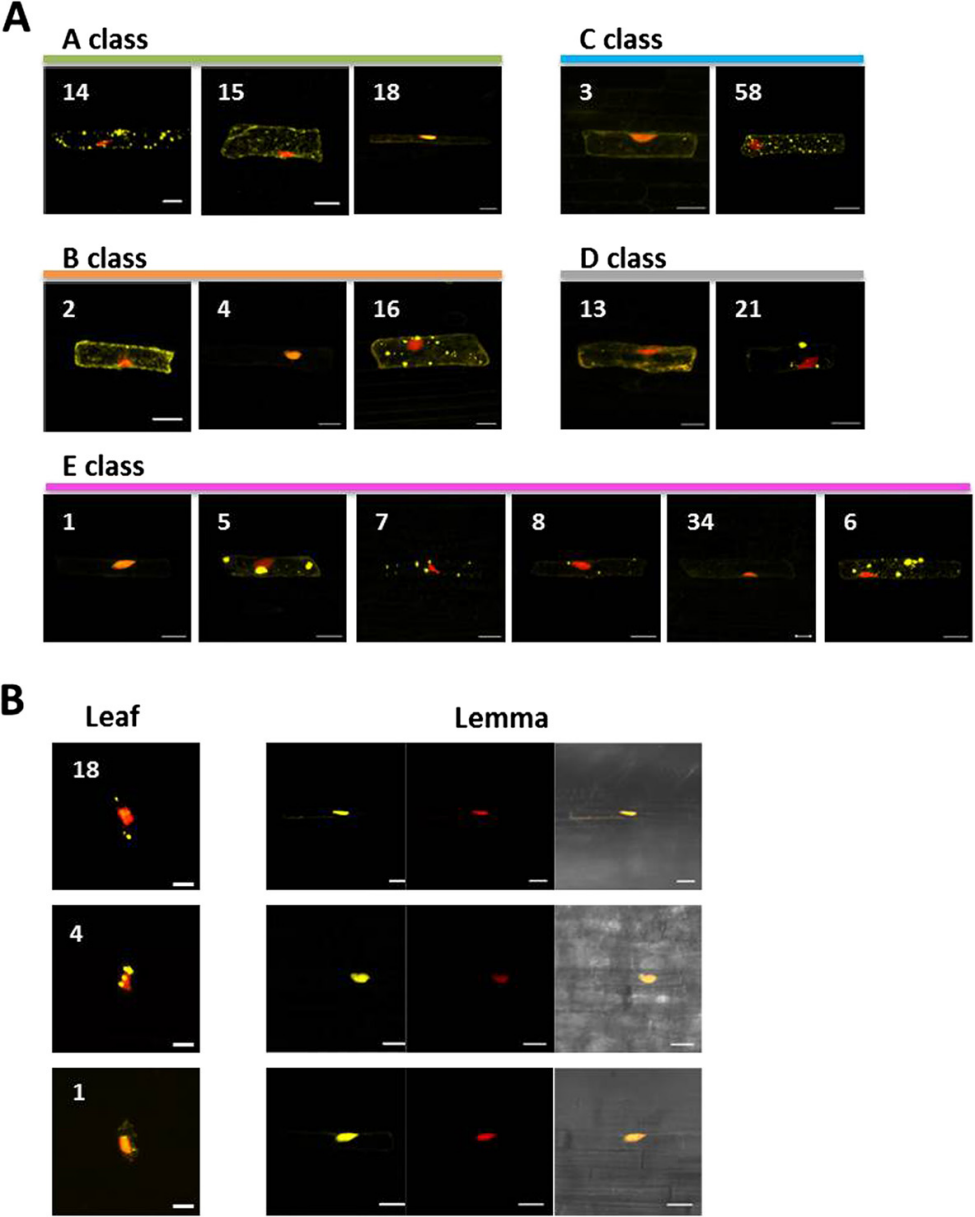
**Subcellular localization of BeMADS fused with fluorescent proteins in *B. edulis *lemmas and leaves. A**. Plasmids harboring a YFP fusion with different BeMADS proteins (yellow signals, the number indicates the gene name) driven by the 35S promoter were transiently expressed in *B. edulis* lemma. These plasmids were delivered by particle bombardment. The NLS domain of VirD2 fused with mCherry was used as the nuclear marker (in red color). Bar = 20 μm. **B**. The subcellular localizations of YFP fusions of BeMADS18, 4 and 1 delivered by particle bombardment into leaf or lemma (yellow signals, Numbers indicate the BeMADS). Red: nuclear marker, VirD2-mCherry signals. Leaf: using leaves as the materials for transient expression. Bar as above.

When lemma was used as bombardment material for subcellular localization, some of the BeMADS proteins were localized to the nucleus (Figure 6A). Interestingly, all of the signal for BeMADS1, 4, and 18 were localized in the nucleus (Figure 6B). In lemma, BeMADS14, 15 (A class), 2, 16 (B class), 58 (C class), 13, 21 (D class), 6 and 7 (E class) did not localize into the nucleus. These results indicated that BeMADS proteins were only translocated into the nucleus in the tissues (lemma) where the gene is normally expressed. Since it is difficult to obtain the bamboo flowers from the field, *in vitro* bamboo flowers were used as target tissues.

**Figure 7:**
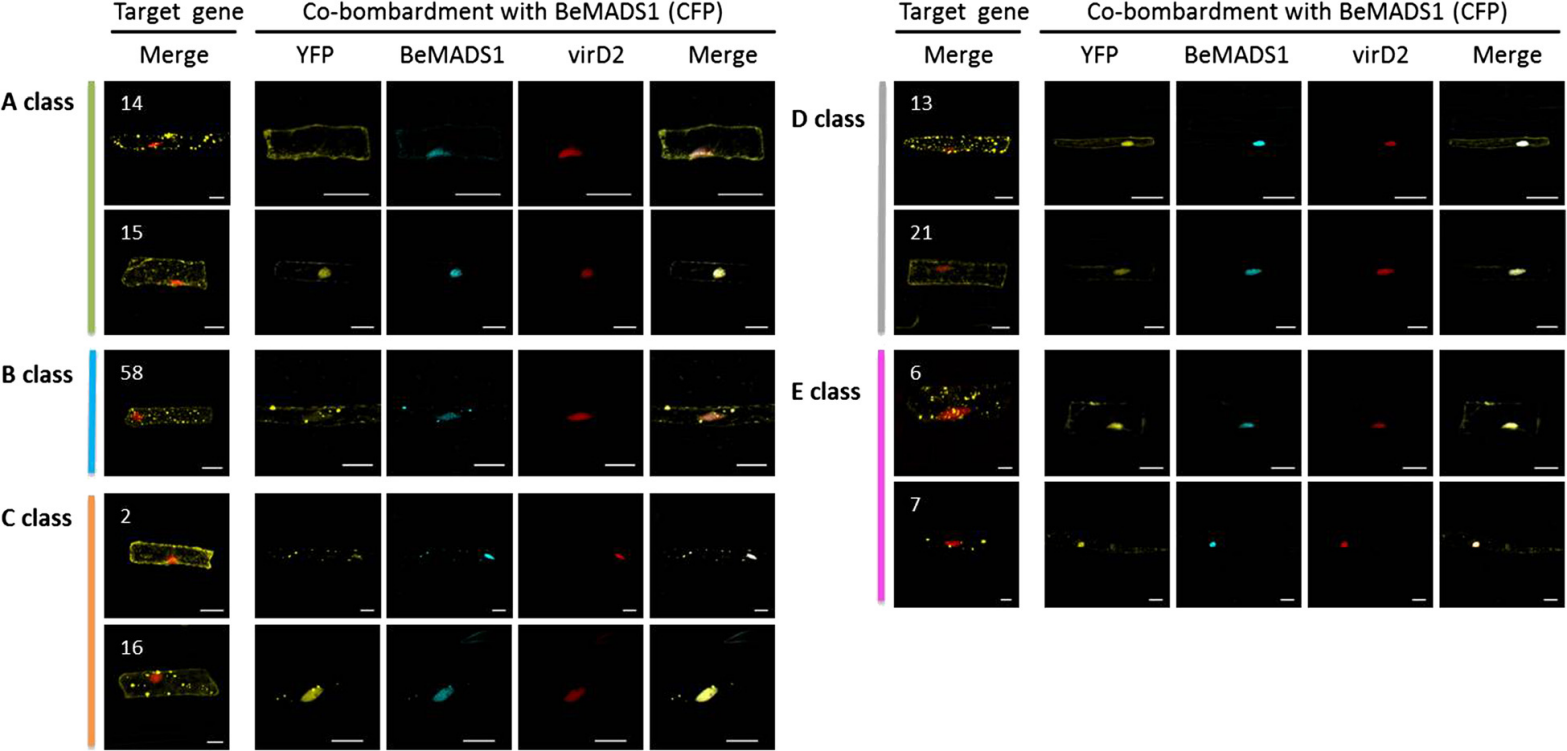
**Nuclear localization of BeMADS proteins during co-transformation with BeMADS1.** Lemmas were used as material for transient transformation by particle bombardment. The tested YFP-BeMADS (numbers in left columns) were co-transformed with BeMADS1-CFP and VirD2-mCherry (nuclear marker). The micrographs in the left column are from Figure 5 and show the localizations of the BeMADS-YFP proteins in *B. edulis* leaves without co-expression of BeMADS1. Bar = 20 μm.

Because MADS proteins form tetramers with other MADS proteins when functioning in floral development [15,61], we hypothesized that some BeMADS proteins do not translocate into the nucleus of lemmas without another MADS protein(s) to assist their import. These 9 *BeMADS* genes (linked to YFP) were co-transformed into lemmas with other nucleus BeMADS proteins (BeMADS1, 4, or 18, as CFP fusions). Our results indicated that only BeMADS1 could facilitate the translocation of these BeMADS proteins into the nucleus in lemma cells (Figure 7; BeMADS4 and 18 not shown). Except BeMADS14 (A class), most of the MADS proteins were translocated to the nucleus, either completely [BeMADS15 (class A), 13 and 21 (class D), and 7 and 6 (class E)] or partially [BeMADS2 and 16 (class B), and BeMADS58 (class C)]. BeMADS14 protein was still in the cytosol when co-transformed with BeMADS1 (Figure 7). The subcellular localization of MADS proteins can be affected by plant growth regulators, growth conditions, like sugar starvation [62], or other protein. For instance, the Arabidopsis MADS SOC1 can interact with AGL24 and then translocate into nucleus to activate *LEAFY* (*LFY*) expression [63]. According to our results, BeMADS1 plays an important role (directly or indirectly) in translocation of other cytosol BeMADS proteins into the nucleus, where they presumably can then function as transcription factors.

These data support previous studies using comprehensive matrix-based screens for petunia and Arabidopsis MADS-box transcription factor interactions, such as FRET (Fluorescence resonance energy transfer)-FLIM (fluorescence lifetime imaging microscopy) imaging and yeast two-, three- or four- hybrid analyses that revealed that MADS-box proteins form multimeric complexes [17,64]. This is the first report on monocot MADS subcellular localization using co-transformation with other proteins or testing different tissue for transient expression.

## Conclusions

Using two different sequencing platforms, a transcriptome database of *B. edulis* was established from plant material grown in tissue culture. The N50 and number of genes in the combined databases are higher than previous bamboo transcriptome results, which used only Illumina methods. The cost of the combined strategy is less than whole genome sequencing. Although the contigs do not contain full length cDNA sequences, these cDNA can be identified by using other public resources, such as moso bamboo whole genome sequences or *B. oldhamii* BAC sequences. To show the usefulness of this strategy, 16 members of the floral development-related MADS gene family were further investigated and cloned. Gene expression and amino acid sequence phylogeny were analyzed and compared to results from other monocot plant species. Since bamboo flowers are difficult to obtain as material for taxonomic and evolutionary studies, these protein sequences may be able to supplement morphological assessments of relatedness and serve as evidence for taxonomy both within Bambusideae and within the wider BEP monocot group.

## Methods

### Plant materials and RNA extraction

The *B. edulis* tissue culture system was established by following our previous protocol [4]. Multiple shoots were incubated in MS medium supplemented with 0.1 mg/l thidiazuron to induce flowering. The inflorescences were subcultured in medium containing 5 mg/l napththalene acetic acid to induce roots, shoots and flowers [65]. The total RNA of these organs were isolated using Trizol reagent (Invitrogen, Carlsbed, CA, USA), following the manufacturer’s instructions. The pooled RNA was used for NGS sequencing.

### The cDNA library preparation, sequencing and assembly on Illumina platform

The cDNA library preparation followed the protocol described previously [8]. The raw sequencing data were filtered to remove low-quality sequences, including ambiguous nucleotides, adaptor sequences, and repeat sequences. The *de novo* transcriptome assemblies of these short reads were performed by the SOAPdenovo program [66] and organized into putative unigenes, which were used for further analysis.

### Roche 454 cDNA library preparation, sequencing and assembly

The cDNA library was constructed using the cDNA Rapid Library Preparation Kit (454 Life Sciences, Roche), starting from 200 ng of mRNA. All steps, including RNA fragmentation, cDNA synthesis, adaptor ligation and product quantification, followed protocols provided by the manufacturer. The resulting cDNA libraries were run on the Roche 454 GS FLX Titanium system. The raw sequence data (.sff) for all reads was obtained from the 454 Genome Sequencer (FLX System). The GS De Novo Assembler software version 2.8 was used for quality/primer trimming and isotig assembling with default parameters, except the "isotig length threshold" was set to 100 bp (default 3) and "Extend low depth overlaps" was enabled. (An isotig is meant to be analogous to an individual transcript.) The output 454 isotigs were then used in further analysis.

### Hybrid transcriptome assembly combining data from 454 and Illumina platforms

A two-phase hybrid assembly approach was performed in order to integrate the 454 platform (producing long reads with homopolymer errors) and Illumina platform (generating huge amount of short reads). The first transcriptome was assembled from the Illumina paired-end reads using a fast short-read assembler (SOAPdenovo) with multiple *k*-mers ranging from 41–51 bp. The second transcriptome was assembled from the 454 reads using a long-read assembler (MIRA) [67] with parameters tuned for 454 sequencing. The third transcriptome was generated from the pre-assembled contigs from the Illumina and 454 data through merger by MIRA with parameters tuned for assembly of Sanger sequencing reads. We aimed to merge concordant contigs assembled from the two platforms into longer contigs and to discard singleton contigs seen by only one platform. The merged MIRA contigs (the hybrid transcriptome) were used in the downstream analysis.

### Functional annotation and classification

Clean reads were obtained by removing the adaptor sequences, empty reads, and the low-quality sequences (with ambiguous sequences ‘N’). Functional annotation of the unigenes was performed by running our assembly against the NCBI non-redundant protein (Nr) database (http://www.ncbi.nlm.nih.gov), the Swiss-Prot protein database (http://www.expasy.ch/sprot), the Kyoto Encyclopedia of Genes and Genomes (KEGG) pathway database (http://www.genome.jp/kegg) and the Cluster of Orthologous Groups (COG) database (http://www.ncbi.nlm.nih.gov/COG) using BLASTX algorithm (E-value threshold: 10^-5^). The proteins that had the highest sequence similarity to our unigenes were used to determine functional annotations. The GO (Gene Ontology) annotations for the unigenes according to component function, biological process or cellular component ontologies were determined by Blast2GO [68]. The WEGO software [69] was used to analyze the GO functional classification for all the unigenes and to understand the distribution of gene functions in *B. edulis* at the macro level. Pathway assignments were made according to KEGG mapping [70]. Sequences were mapped to the KEGG biochemical pathways according to the Enzyme Commission (EC) distribution within the pathway database.

### Phylogenetic analysis of BeMADS proteins

The MADS amino acid sequences from bamboo identified in this report and from other plant species were obtained from the NCBI database (http://www.ncbi.nlm.nih.gov/). Comparison with the bamboo MADS proteins was conducted by aligning all sequences in FASTA format using CLUSTAL W [71]. Multiple sequence alignment, phylogenetic, and molecular evolutionary analyses were conducted using MEGA software version 4 [72]. The distance matrices for the aligned sequences with all gaps ignored were calculated using the Kimura two-parameter method. Further molecular phylogenetic analyses used the neighbor-joining (NJ) method after alignment [73]. One thousand bootstrap resampling replicates were conducted to estimate support for the clades. Arabidopsis FLC genes were used as the root [74].

### Real-time quantitative reverse transcription (qRT)-PCR

Plant tissues (Additional file 5) from *in vitro* cultures were excised for organ-specific RNA extraction, which was performed using Trizol as described above. RNA (2 μg) was reversely transcribed using Superscript III Reverse Transcriptase kit (Invitrogen) according to the manufacturer’s instructions. The expression level of a target gene was detected with SYBR Green real-time PCR on Rotor-Gene Q real-time thermocyclers (Corbett Research, Australia). Data were analyzed using the Rotor-Gene Q software version 2.0 (Corbett Research) and Microsoft Excel (Microsoft, USA). Tubulin was used as the internal control. Experiments were performed for three biological repeats in triplicate. The primers are given in Additional file 6.

### *In situ* hybridization

*In situ* hybridization was performed as previously described [75]. Tissue sections after *in situ* hybridization were photographed on a Zeiss Axio Scope A1 microscope equipped with an Axio- Cam HRc camera (Zeiss, Germany).

### Subcellular localization of BeMADS-YFP

Full-length cDNAs were amplified using PCR incorporating *B. edulis* cDNA as template (primer information, Additional file 6). Products were cloned into pDONR221 by Gateway BP Clonase II Enzyme Mix (Invitrogen) and into p2GWF7 (nYFP) using Gateway LR Clonase II Enzyme Mix (Invitrogen, [76]). The plasmids (2.5 μg) were isolated and transformed into *B. edulis* leaves or lemmas using bombardment transformation. The transformed tissues were incubated overnight before observation on a Zeiss LSM 510 META laser-scanning confocal microscope using an LD C-Apochromat40×/1.1 W objective lens [33].

### Availability of supporting data

The Next generation sequencing data from this study were deposited at the Sequence Read Archive (SRP043102; http://www.ncbi.nlm.nih.gov/sra/?term=SRP043102)

## Competing interests

The authors declare that they have no competing interests.

## Authors’ contributions

CTH, WJC and DCL performed the preparation of RNA, cDNA, qRT-PCR and subcellular localization analyses. MLC performed the phylogenetic tree analysis. SSK participated in the *in situ* hybridization studies. YTH, JJWC, JLY and XPG performed bioinformatic analyses. MCS, MLC and JJY contributed equally to the design and helped draft the manuscript. CSL prepare the manuscript and directed the whole study. All authors read and approved the final manuscript.

## Supplementary Material

Additional file 1 The sequences of three databases. Click here for file

Additional file 2 **Annotation statistics from three *B. edulis *** **transcriptome datasets.** Statistics of annotation from three *B. edulis* transcriptome datasets. The last column indicates the percentage of sequences which can be annotated in at least one method. Click here for file

Additional file 3 **Unigene metabolic pathway analysis from three *B. edulis *** **transcriptome datasets.** Unigene metabolic pathway analysis from three *B. edulis* transcriptome datasets. **(A)** 454 dataset. **(B)** Illumina dataset. **(C)** Hybrid dataset. These sequences were analysis by Kyoto Encyclopedia of Genes and Genomes (KEGG) pathway database. Click here for file

Additional file 4 **Accession numbers of *B. edulis *** **floral development-related MADS genes.** The accession numbers of *B. edulis* flower development-related MADS genes as deposited into the NCBI database. Click here for file

Additional file 5 **Flower material for qRT-PCR.** The flower material for qRT-PCR. (Left) Each spikelet in *B. edulis* has multiple florets. The florets were numbered 1–5, young to old. Bar = 1 mm. (Right). The mature florets are enclosed by two bracts called the palaea (Pa) and lemma (Le). The perianth of each floret is represented by two transparent scales called lodicules (Lo). There are generally three anthers (An) and a pistil (Pi) with two hairy stigmatic lobes. Bar = 1 mm. Click here for file

Additional file 6 **Primer list.** The primer list in this study. Primers for full length genes were used in PCR with DNA from the *Bambusa edulis* BAC library. Click here for file

Additional file 7 **Subcellular localization of BeMADS fused with fluorescent proteins in *B. edulis *** **leaves.** Subcellular localization of BeMADS fused with fluorescent proteins in *B. edulis* leaves. Plasmids harboring a YFP fusion with different BeMADS proteins (Yellow signals, the number indicates the gene name) driven by the 35S promoter were transiently expressed in *B. edulis* leaves. The functional classification according to A/B/C/D/E class are indicated at the top of the panels. These plasmids were delivered by particle bombardment. The NLS domain of VirD2 fused with mCherry was used as the nuclear marker (in red color). Only the merged images are shown. Bar = 20 μm. Click here for file

## References

[B1] Li Z Denich M Is Shennongjia a suitable site for reintroducing giant panda: an appraisal on food supply Environmentalist 2004 24 165 170

[B2] Xiong W The present and future situation of bamboo industry in Japan Bamboo Res 1982 1 92 93

[B3] Nadgauda RS Parasharami VA Mascarenhas AF Precocious flowering and seeding behavior in tissue cultured bamboos Nature 1990 344 335 336

[B4] Lin CS Chang WC Micropropagation of *Bambusa edulis* through nodal explants of field-grown culms and flowering of regenerated plantlets Plant Cell Rep 1998 17 617 620 10.1007/s002990050453 30736514

[B5] Lin CS Lin CC Chang WC *In vitro* flowering of *Bambusa edulis* and subsequent plantlet survival Plant Cell Tiss Org 2003 72 71 78

[B6] Lin CS Lai YH Sun CW Liu NT Tsay HS Chang WC Chen JJW Identification of ESTs differentially expressed in green and albino mutant bamboo (*Bambusa edulis*) by suppressive subtractive hybridization (SSH) and microarray analysis Plant Cell Tiss Org 2006 86 169 175

[B7] Liu NT Wu FH Tsay HS Chang WC Lin CS Establishment of a cDNA library from *Bambusa edulis* Murno in vitro-grown shoots Plant Cell Tiss Org 2008 95 21 27

[B8] Chou ML Shih MC Chan MT Liao SY Hsu CT Haung YT Chen JJW Liao DC Wu FH Lin CS Global transcriptome analysis and identification of a *CONSTANS*-like gene family in the orchid *Erycina pusilla* Planta 2013 237 1425 1441 23417646 10.1007/s00425-013-1850-z

[B9] Metzker ML Sequencing technologies - the next generation Nat Rev Genet 2010 11 31 46 19997069 10.1038/nrg2626

[B10] Liu M Qiao G Jiang J Yang H Xie L Xie J Zhuo R Transcriptome sequencing and *De Novo* analysis for ma bamboo (*Dendrocalamus latiflorus* Munro) using the illumina platform PLoS One 2012 7 e46766 23056442 10.1371/journal.pone.0046766 PMC3463524

[B11] Zhang XM Zhao L Larson-Rabin Z Li DZ Guo ZH *De Novo* sequencing and characterization of the floral transcriptome of *Dendrocalamus latiflorus* (Poaceae: Bambusoideae) PLoS One 2012 7 8 e42082 22916120 10.1371/journal.pone.0042082 PMC3419236

[B12] Peng Z Lu Y Li L Zhao Q Feng Q Gao Z Lu H Hu T Yao N Liu K Li Y Fan D Guo Y Li W Lu Y Weng Q Zhou C Zhang L Huang T Zhao Y Zhu C Liu X Yang X Wang T Miao K Zhuang C Cao X Tang W Liu G Liu Y Chen J Liu Z Yuan L Liu Z Huang X Lu T Fei B Ning Z Han B Jiang Z The draft genome of the fast-growing non-timber forest species moso bamboo (*Phyllostachys heterocycla*) Nat Genet 2013 45 456 461 23435089 10.1038/ng.2569

[B13] Ning CQ Dai QH Cross breeding of *Bambusa pervariabilis* x *Dendrocalamopsis grandis* Guangxi Forestry Sci 1995 4 167 168

[B14] Theissen G Melzer R Molecular mechanisms underlying origin and diversification of the angiosperm flower Ann Bot-London 2007 100 603 619 10.1093/aob/mcm143 PMC2533597 17670752

[B15] Coen ES Meyerowitz EM The war of the whorls: genetic interactions controlling flower development Nature 1991 353 31 37 1715520 10.1038/353031a0

[B16] Pelaz S Ditta GS Baumann E Wisman E Yanofsky MF B and C floral organ identity functions require *SEPALLATA* MADS-box genes Nature 2000 405 200 203 10821278 10.1038/35012103

[B17] Favaro R Pinyopich A Battaglia R Kooiker M Borghi L Ditta G Yanofsky MF Kater MM Colombo L MADS-box protein complexes control carpel and ovule development in Arabidopsis Plant Cell 2003 15 2603 2611 14555696 10.1105/tpc.015123 PMC280564

[B18] Pinyopich A Ditta GS Savidge B Liljegren SJ Baumann E Wisman E Yanofsky MF Assessing the redundancy of MADS-box genes during carpel and ovule development Nature 2003 424 85 88 12840762 10.1038/nature01741

[B19] Barker NP Clark LG Davis JI Duvall MR Guala GF Hsiao C Kellogg EA Linder HP Mason-Gamer RJ Mathews SY Simmons MP Soreng RJ Spangler RE Grass Phylogeny Working Group Phylogeny and subfamilial classification of the grasses (Poaceae) Ann Missouri Bot Gard 2001 88 373 457

[B20] Preston JC Kellogg EA Conservation and divergence of *APETALA1*/*FRUITFULL*-like gene function in grasses: evidence from gene expression analyses Plant J 2007 52 69 81 17666026 10.1111/j.1365-313X.2007.03209.x

[B21] Nagasawa N Miyoshi M Sano Y Satoh H Hirano H Sakai H Nagato Y *SUPERWOMAN1* and *DROOPING LEAF* genes control floral organ identity in rice Development 2003 130 705 718 12506001 10.1242/dev.00294

[B22] Fornara F Parenicova L Falasca G Pelucchi N Masiero S Ciannamea S Lopez-Dee Z Altamura MM Colombo L Kater MM Functional characterization of *OsMADS18*, a member of the *AP1*/*SQUA* subfamily of MADS box genes Plant Physiol 2004 135 2207 2219 15299121 10.1104/pp.104.045039 PMC520791

[B23] Kater MM Dreni L Colombo L Functional conservation of MADS-box factors controlling floral organ identity in rice and Arabidopsis J Exp Bot 2006 57 3433 3444 16968881 10.1093/jxb/erl097

[B24] Ambrose BA Lerner DR Ciceri P Padilla CM Yanofsky MF Schmidt RJ Molecular and genetic analyses of the silky1 gene reveal conservation in floral organ specification between eudicots and monocots Mol Cell 2000 5 569 579 10882141 10.1016/s1097-2765(00)80450-5

[B25] Yao SG Ohmori S Kimizu M Yoshida H Unequal genetic redundancy of rice *PISTILLATA* orthologs, *OsMADS2* and *OsMADS4*, in lodicule and stamen development Plant Cell Physiol 2008 49 853 857 18378529 10.1093/pcp/pcn050

[B26] Yamaguchi T Lee DY Miyao A Hirochika H An GH Hirano HY Functional diversification of the two C-class MADS box genes *OSMADS3* and *OSMADS58* in *Oryza sativa* Plant Cell 2006 18 15 28 16326928 10.1105/tpc.105.037200 PMC1323481

[B27] Cui R Han J Zhao S Su K Wu F Du X Xu Q Chong K Theissen G Meng Z Functional conservation and diversification of class E floral homeotic genes in rice (*Oryza sativa*) Plant J 2010 61 767 781 20003164 10.1111/j.1365-313X.2009.04101.x

[B28] Li H Liang W Hu Y Zhu L Yin C Xu J Dreni L Kater MM Zhang D Rice *MADS6* interacts with the floral homeotic genes *SUPERWOMAN1*, *MADS3*, *MADS58*, *MADS13*, and *DROOPING LEAF* in specifying floral organ identities and meristem fate Plant Cell 2011 23 2536 2552 21784949 10.1105/tpc.111.087262 PMC3226212

[B29] Tian B Chen YY Yan YX Li DZ Isolation and ectopic expression of a bamboo MADS-box gene Chinese Sci Bull 2005 50 217 224

[B30] Tian B Chen YY Li DZ Yan YX Cloning and characterization of a bamboo *LEAFY HULL STERILE1* homologous gene DNA Seq 2006 17 143 151 17076257 10.1080/10425170600699877

[B31] Lin EP Peng HZ Jin QY Deng MJ Li T Xiao XC Hua XQ Wang KH Bian HW Han N Identification and characterization of two bamboo (*Phyllostachys praecox*) AP1/SQUA-like MADS-box genes during floral transition Planta 2009 231 109 120 19855996 10.1007/s00425-009-1033-0

[B32] Su CL Chao YT Chang YCA Chen WC Chen CY Lee AY Hwa KT Shih MC *De Novo* assembly of expressed transcripts and global analysis of the *Phalaenopsis aphrodite* transcriptome Plant Cell Physiol 2011 52 1501 1514 21771864 10.1093/pcp/pcr097

[B33] Hsu CT Liao DC Wu FH Liu NT Shen SC Chou SJ Tung SY Yang CH Chan MT Lin CS Integration of molecular biology tools for identifying promoters and genes abundantly expressed in flowers of *Oncidium* Gower Ramsey BMC Plant Biol 2011 11 60 21473751 10.1186/1471-2229-11-60 PMC3079641

[B34] Lin CS Chen JJW Huang YT Hsu CT Lu HC Chou ML Chen LC Ou CI Liao DC Yeh YY Chang SB Shen SC Wu FH Shih MC Chan MT Catalog of *Erycina pusilla* miRNA and categorization of reproductive phase-related miRNAs and their target gene families Plant Mol Biol 2013 82 193 204 23575662 10.1007/s11103-013-0055-y

[B35] Pan IC Liao DC Wu FH Daniell H Singh ND Chang C Shih MC Chan MT Lin CS Complete chloroplast genome sequence of an orchid model plant candidate: *Erycina pusilla* apply in tropical oncidium breeding PLoS One 2012 7 e34738 22496851 10.1371/journal.pone.0034738 PMC3319614

[B36] Wu FH Kan DP Lee SB Daniell H Lee YW Lin CC Lin NS Lin CS Complete nucleotide sequence of *Dendrocalamus latiflorus* and *Bambusa oldhamii* chloroplast genomes Tree Physiol 2009 29 847 856 19324693 10.1093/treephys/tpp015 PMC2762994

[B37] Zhang T Zhang X Hu S Yu J An efficient procedure for plant organellar genome assembly, based on whole genome data from the 454 GS FLX sequencing platform Plant Methods 2011 7 38 22126655 10.1186/1746-4811-7-38 PMC3248859

[B38] Arora R Agarwal P Ray S Singh AK Singh VP Tyagi AK Kapoor S MADS-box gene family in rice: genome-wide identification, organization and expression profiling during reproductive development and stress BMC Genomics 2007 8 242 17640358 10.1186/1471-2164-8-242 PMC1947970

[B39] Zhao Y Li X Chen W Peng X Cheng X Zhu S Cheng B Whole-genome survey and characterization of MADS-box gene family in maize and sorghum Plant Cell Tiss Org 2011 105 159 173

[B40] Paollacci AR Tanzarella OA Porceddu E Varotto S Ciaffi M Molecular and phylogenetic analysis of MADS-box genes of MIKC type and chromosome location of *SEP*-like genes in wheat (*Triticum aestivum* L.) Mol Genet Genomics 2007 278 689 708 17846794 10.1007/s00438-007-0285-2

[B41] Malcomber ST Kellogg EA *SEPALLATA* gene diversification: brave new whorls Trends Plant Sci 2005 10 427 435 16099195 10.1016/j.tplants.2005.07.008

[B42] Münster T Wingen LU Faigl W Werth S Saedler H Theissen G Characterization of three *GLOBOSA*-like MADS-box genes from maize: evidence for ancient paralogy in one class of floral homeotic B-function genes of grasses Gene 2001 262 1 13 11179662 10.1016/s0378-1119(00)00556-4

[B43] Rounsley SD Ditta GS Yanofsky MF Diverse roles for MADS box genes in Arabidopsis development Plant Cell 1995 7 1259 1269 7549482 10.1105/tpc.7.8.1259 PMC160949

[B44] Dreni L Jacchia S Fornara F Fornari M Ouwerkerk PBF An G Colombo L Kater MM The D-lineage MADS-box gene *OsMADS13* controls ovule identity in rice Plant J 2007 52 690 699 17877710 10.1111/j.1365-313X.2007.03272.x

[B45] Ciaffi M Paolacci AR Tanzarella OA Porceddu E Molecular aspects of flower development in grasses Sex Plant Reprod 2011 24 247 282 21877128 10.1007/s00497-011-0175-y

[B46] Christensen AR Malcomber ST Duplication and diversification of the *LEAFY HULL STERILE1* and *Oryza sativa MADS5 SEPALLATA* lineages in graminoid Poales EvoDevo 2012 3 4 22340849 10.1186/2041-9139-3-4 PMC3305426

[B47] Li R Zhu H Ruan J Qian W Fang X Shi Z Li Y Li S Shan G Kristiansen K Li S Yang H Wang J Wang J *De novo* assembly of human genomes with massively parallel short read sequencing Genome Res 2010 20 265 272 20019144 10.1101/gr.097261.109 PMC2813482

[B48] Ohmori S Kimizu M Sugita M Miyao A Hirochika H Uchida E Nagato Y Yoshida H *MOSAIC FLORAL ORGANS1*, an *AGL6-*Like MADS box gene, regulates floral organ identity and meristem fate in rice Plant Cell 2009 21 3008 3025 19820190 10.1105/tpc.109.068742 PMC2782282

[B49] Zahn LM King HZ Leebens-Mack JH Kim S Soltis PS Landherr LL Soltis DE dePamphilis CW Ma H The evolution of the *SEPALLATA* subfamily of MADS-Box genes: a preangiosperm origin with multiple duplications throughout angiosperm history Genetics 2005 169 2209 2223 15687268 10.1534/genetics.104.037770 PMC1449606

[B50] Gao X Liang W Yin C Ji S Wang H Su X Guo C Kong H Xue H Zhang D The *SEPALLATA*-like gene *OsMADS34* is required for rice inflorescence and spikelet development Plant Physiol 2010 153 728 740 20395452 10.1104/pp.110.156711 PMC2879775

[B51] Pelucchi N Fornara F Favalli C Masiero S Lago C Pe ME Colombo L Kater MM Comparative analysis of rice MADS-box genes expressed during flower development Sex Plant Reprod 2002 15 113 122

[B52] Kyozuka J Kobayashi T Morita M Shimamoto K Spatially and temporally regulated expression of rice MADS box genes with similarity to Arabidopsis class A, B and C genes Plant Cell Physiol 2000 41 710 718 10945340 10.1093/pcp/41.6.710

[B53] Goto K Meyerowitz EM Function and regulation of the Arabidopsis floral homeotic gene *PISTILLATA* Genes Dev 1994 8 1548 1560 7958839 10.1101/gad.8.13.1548

[B54] Lopez-Dee ZP Wittich P Pe ME Rigola D Del Buono I Gorla MS Kater MM Colombo L *OsMADS13*, a novel rice MADS-box gene expressed during ovule development Dev Genet 1999 25 237 244 10528264 10.1002/(SICI)1520-6408(1999)25:3<237::AID-DVG6>3.0.CO;2-L

[B55] Schmidt RJ Veit B Mandel MA Mena M Hake S Yanofsky MF Identification and molecular characterization of *ZAG1*, the maize homolog of the Arabidopsis floral homeotic gene *AGAMOUS* Plant Cell 1993 5 729 737 8103379 10.1105/tpc.5.7.729 PMC160311

[B56] Ditta G Pinyopich A Robles P Pelaz S Yanofsky MF The *SEP4* gene of *Arabidopsis thaliana* functions in floral organ and meristem identity Curr Biol 2004 14 1935 1940 15530395 10.1016/j.cub.2004.10.028

[B57] Pelaz S Tapia-Lopez R Alvarez-Buylla ER Yanofsky MF Conversion of leaves into petals in Arabidopsis Curr Biol 2001 11 182 184 11231153 10.1016/s0960-9822(01)00024-0

[B58] Theissen G Saedler H Plant biology - floral quartets Nature 2001 409 469 471 11206529 10.1038/35054172

[B59] Prasad K Sriram P Kumar CS Kushalappa K Vijayraghavan U Ectopic expression of rice *OsMADS1* reveals a role in specifying the lemma and palea, grass floral organs analogous to sepals Dev Genes Evol 2001 211 281 290 11466523 10.1007/s004270100153

[B60] Reinheimer R Kellogg EA Evolution of *AGL6*-like MADS box genes in grasses (Poaceae): ovule expression is ancient and palea expression is new Plant Cell 2009 21 2591 2605 19749151 10.1105/tpc.109.068239 PMC2768931

[B61] Krizek BA Fletcher JC Molecular mechanisms of flower development: an armchair guide Nat Rev Genet 2005 6 688 698 16151374 10.1038/nrg1675

[B62] Hong YF Ho THD Wu CF Ho SL Yeh RH Lu CA Chen PW Yu LC Chao AL Yu SM Convergent starvation signals and hormone crosstalk in regulating nutrient mobilization upon germination in cereals Plant Cell 2012 24 2857 2873 22773748 10.1105/tpc.112.097741 PMC3426119

[B63] Lee J Oh M Park H Lee I SOC1 translocated to the nucleus by interaction with AGL24 directly regulates LEAFY Plant J 2008 55 832 843 18466303 10.1111/j.1365-313X.2008.03552.x

[B64] de Folter S Immink RG Kieffer M Pařenicová L Henz SR Weigel D Busscher M Kooiker M Colombo L Kater MM Daviesb B Angenent GC Comprehensive interaction map of the Arabidopsis MADS box transcription factors Plant Cell 2005 17 1424 1433 15805477 10.1105/tpc.105.031831 PMC1091765

[B65] Lin CS Lin CC Chang WC Shoot regeneration, re-flowering and post flowering survival in bamboo inflorescence culture Plant Cell Tiss Org 2005 82 243 249

[B66] Luo R Liu B Xie Y Li Z Huang W Yuan J He G Chen Y Pan Q Liu Y Tang J Wu G Zhang H Shi Y Liu Y Yu C Wang B Lu Y Han C Cheung DW Yiu S-M Peng S Xiaoqian Z Liu G Liao X Li Y Yang H Wang J Lam T-W Wang J SOAPdenovo2: an empirically improved memory-efficient short-read de novo assembler Gigascience 2012 1 1 18 23587118 10.1186/2047-217X-1-18 PMC3626529

[B67] Chevreux B Pfisterer T Drescher B Driesel AJ Müller WE Wetter T Suhai S Using the miraEST assembler for reliable and automated mRNA transcript assembly and SNP detection in sequenced ESTs Genome Res 2004 14 1147 1159 15140833 10.1101/gr.1917404 PMC419793

[B68] Conesa A Götz S García-Gómez JM Terol J Talón M Robles M Blast2GO: a universal tool for annotation, visualization and analysis in functional genomics research Bioinformatics 2005 21 3674 3676 16081474 10.1093/bioinformatics/bti610

[B69] Ye J Fang L Zheng H Zhang Y Chen J Zhang Z Wang J Li S Li R Bolund L Wang J WEGO: a web tool for plotting GO annotations Nucleic Acids Res 2006 34 W293 W297 16845012 10.1093/nar/gkl031 PMC1538768

[B70] Kanehisa M Araki M Goto S Hattori M Hirakawa M Itoh M Katayama T Kawashima S Okuda S Tokimatsu T Yamanishi Y KEGG for linking genomes to life and the environment Nucleic Acids Res 2008 36 D480 D484 18077471 10.1093/nar/gkm882 PMC2238879

[B71] Thompson JD Higgins DG Gibson TJ CLUSTAL W: improving the sensitivity of progressive multiple sequence alignment through sequence weighting, position-specific gap penalties and weight matrix choice Nucleic Acids Res 1994 22 4673 4680 7984417 10.1093/nar/22.22.4673 PMC308517

[B72] Tamura K Dudley J Nei M Kumar S MEGA4: Molecular evolutionary genetics analysis (MEGA) software version 4.0 Mol Biol Evol 2007 24 1596 1599 17488738 10.1093/molbev/msm092

[B73] Altschul SF Gish W Miller W Myers EW Lipman DJ Basic local alignment search tool J Mol Biol 1990 215 403 410 2231712 10.1016/S0022-2836(05)80360-2

[B74] Michaels SD Amasino RM *FLOWERING LOCUS C* encodes a novel MADS domain protein that acts as a repressor of flowering Plant Cell 1999 11 949 956 10330478 10.1105/tpc.11.5.949 PMC144226

[B75] Lin HY Chen JC Wei MJ Lien YC Li HH Ko SS Liu ZH Fang SC Genome-wide annotation, expression profiling, and protein interaction studies of the core cell-cycle genes in *Phalaenopsis aphrodite* Plant Mol Biol 2014 84 203 226 24222213 10.1007/s11103-013-0128-y PMC3840290

[B76] Karimi M Inze D Depicker A GATEWAY vectors for Agrobacterium-mediated plant transformation Trends Plant Sci 2002 7 193 195 11992820 10.1016/s1360-1385(02)02251-3

