# Supplementary material for: BeMADS1 is a key to delivery MADSs into nucleus in reproductive tissues-De novo characterization of Bambusa edulis transcriptome and study of MADS genes in bamboo floral development
Source: BMC Plant Biol. 2014 Jul 2;14:179. doi: 10.1186/1471-2229-14-179 (PMC4087239; doi:10.1186/1471-2229-14-179)
Supplement: Additional file 2 — Annotation statistics from three B. edulis transcriptome datasets. Statistics of annotation from three B. edulis transcriptome datasets. The last column indicates the percentage of sequences which can be annotated in at least one method. [file 1471-2229-14-179-S2.docx]

**Additional file 2.**

Statistics of annotation results from three *B. edulis* transcriptome datasets.

| Sequence File | Sequence no. | Nr | Swiss-Prot | KEGG | COG | GO | ALL | ALL/ Sequence no. x 100% |
| --- | --- | --- | --- | --- | --- | --- | --- | --- |
| 454 | 15,117 | 11,583 | 7,961 | 11,504 | 9,347 | 4,030 | 11,659 | 77.13% |
| Illumina | 54,830 | 39,048 | 24,129 | 38,757 | 29,654 | 11,251 | 39,325 | 71.72% |
| Hybrid | 8,241 | 7,120 | 5,258 | 7,095 | 6,158 | 2,930 | 7,144 | 86.69% |
