# Supplementary material for: BeMADS1 is a key to delivery MADSs into nucleus in reproductive tissues-De novo characterization of Bambusa edulis transcriptome and study of MADS genes in bamboo floral development
Source: BMC Plant Biol. 2014 Jul 2;14:179. doi: 10.1186/1471-2229-14-179 (PMC4087239; doi:10.1186/1471-2229-14-179)

Additional file 3.

Unigene metabolic pathway analysis from three *B. edulis* transcriptome datasets.

1. 454 dataset


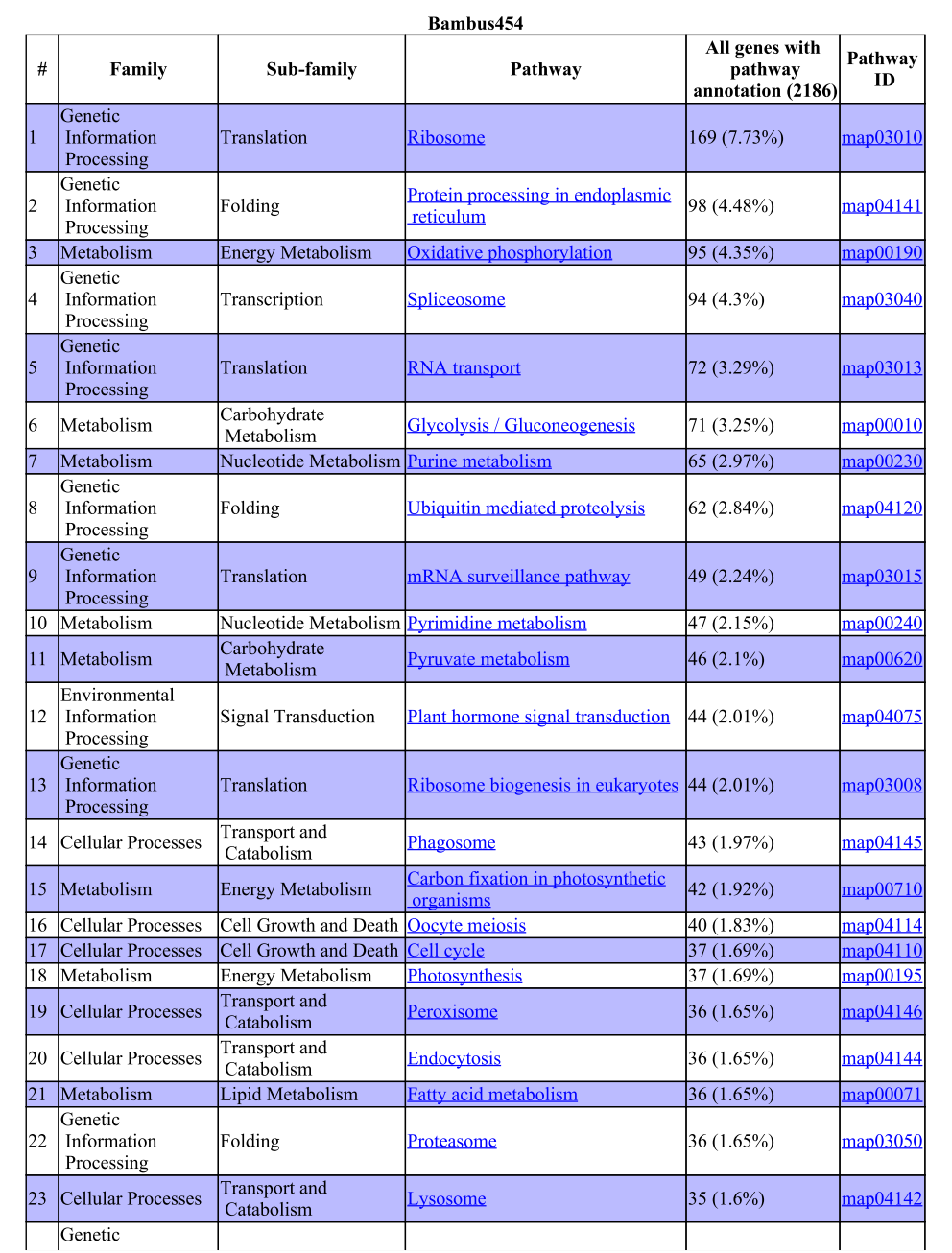


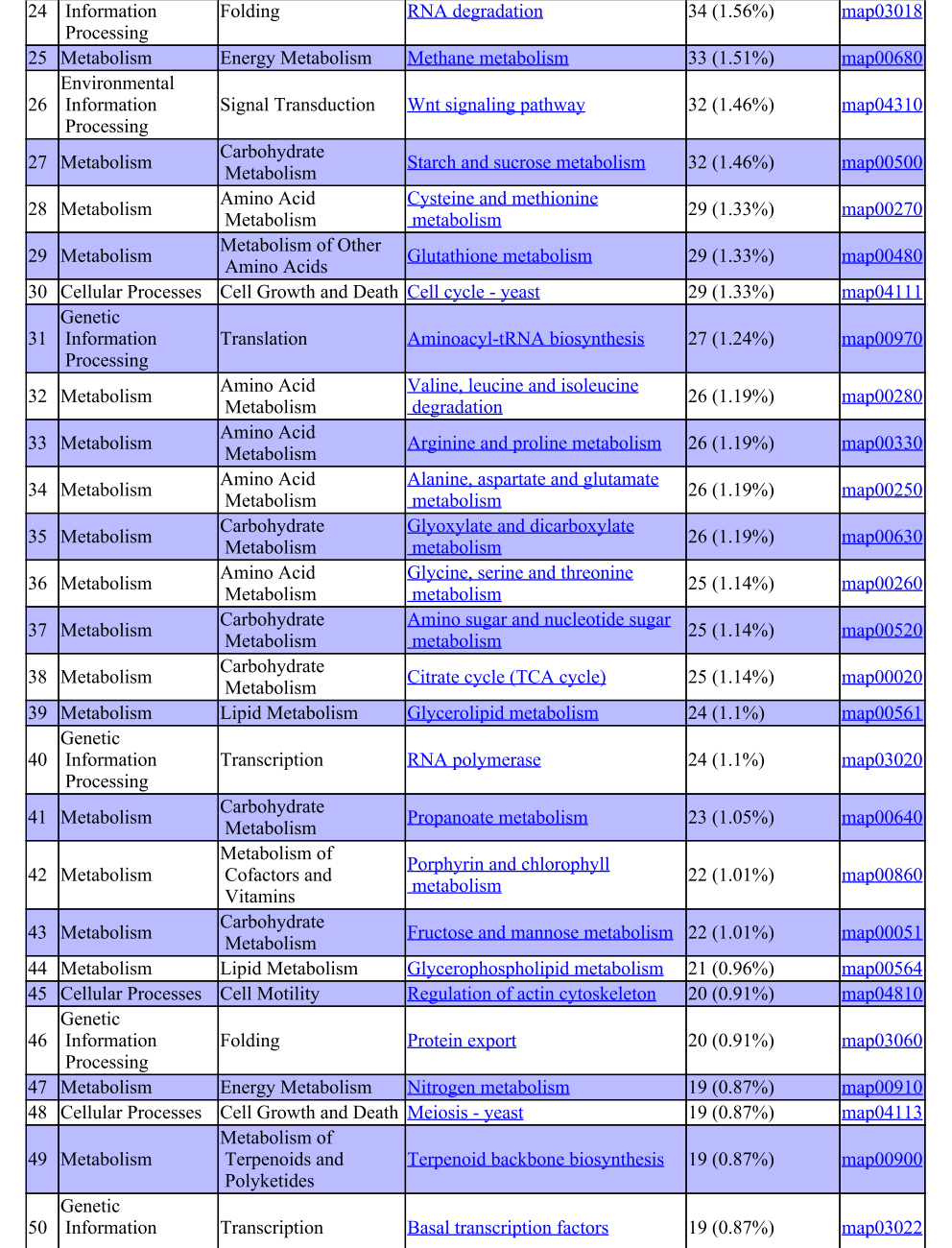


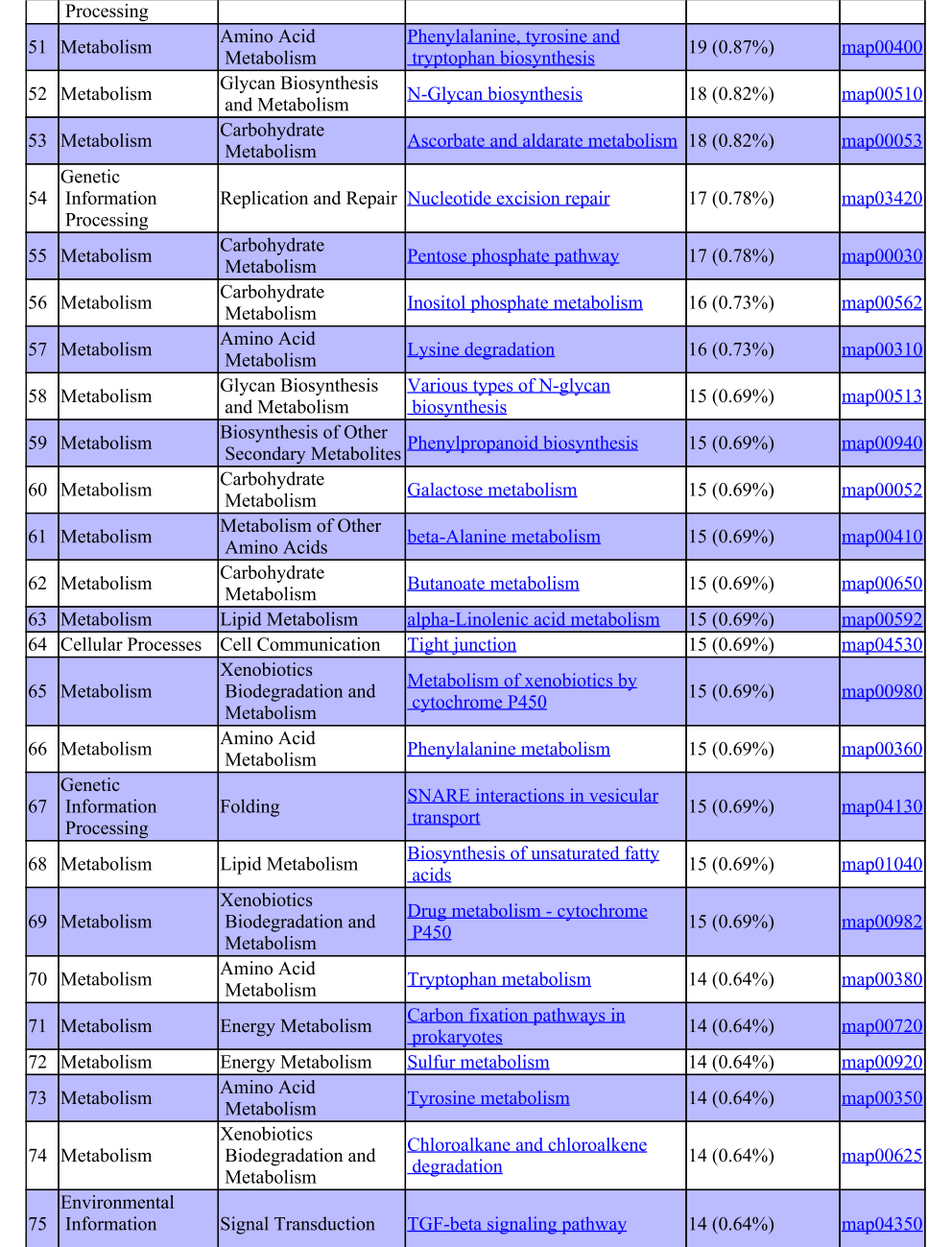


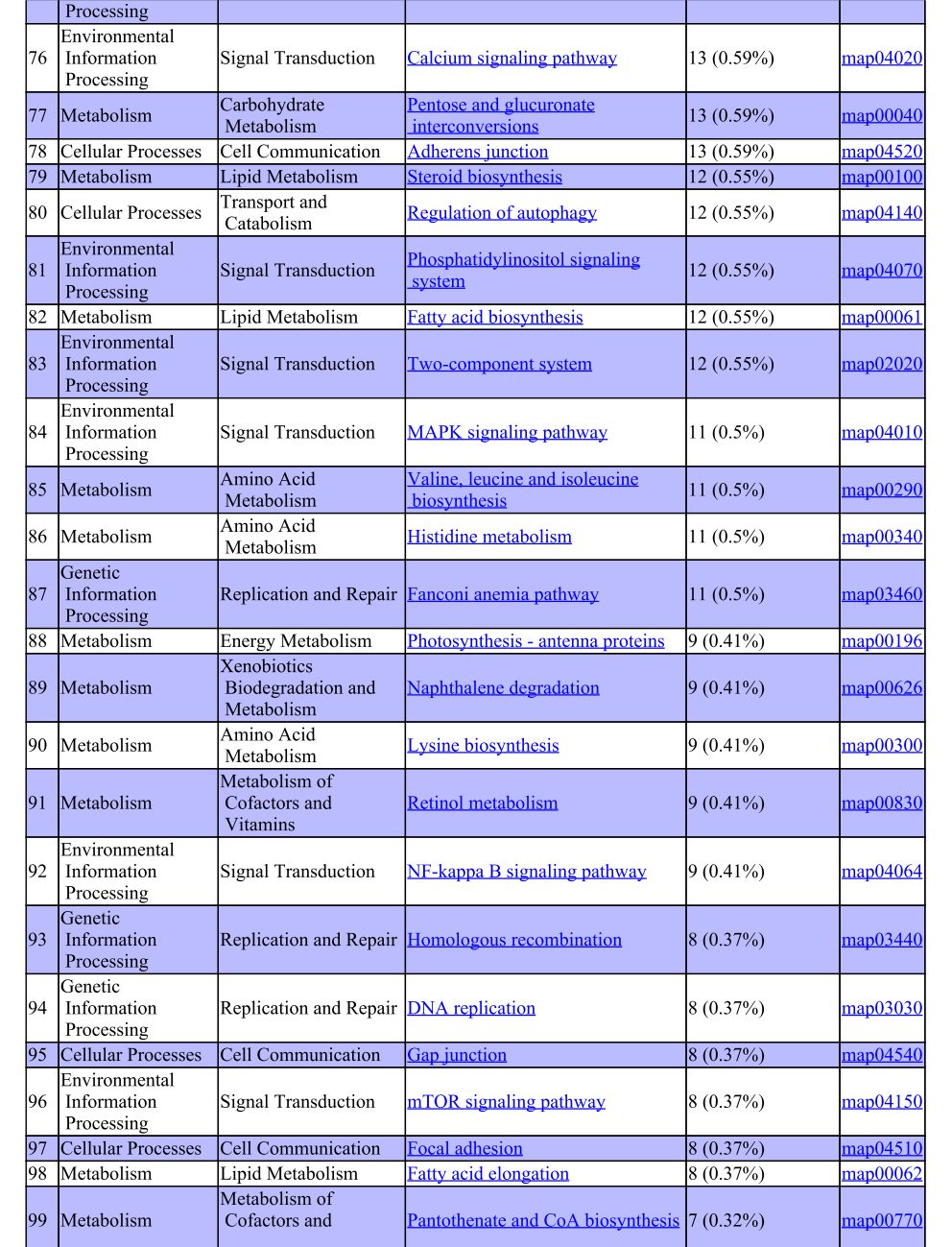


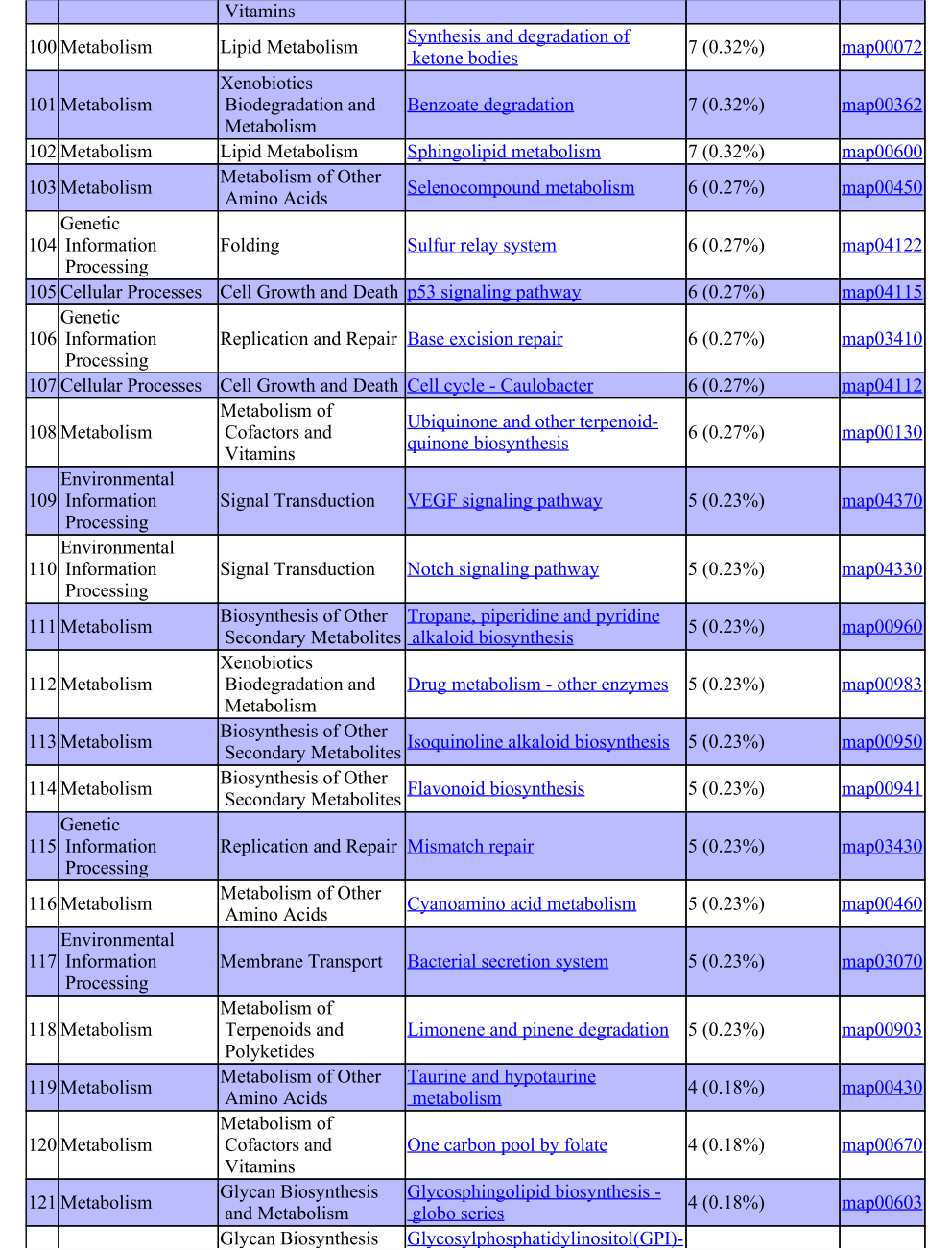


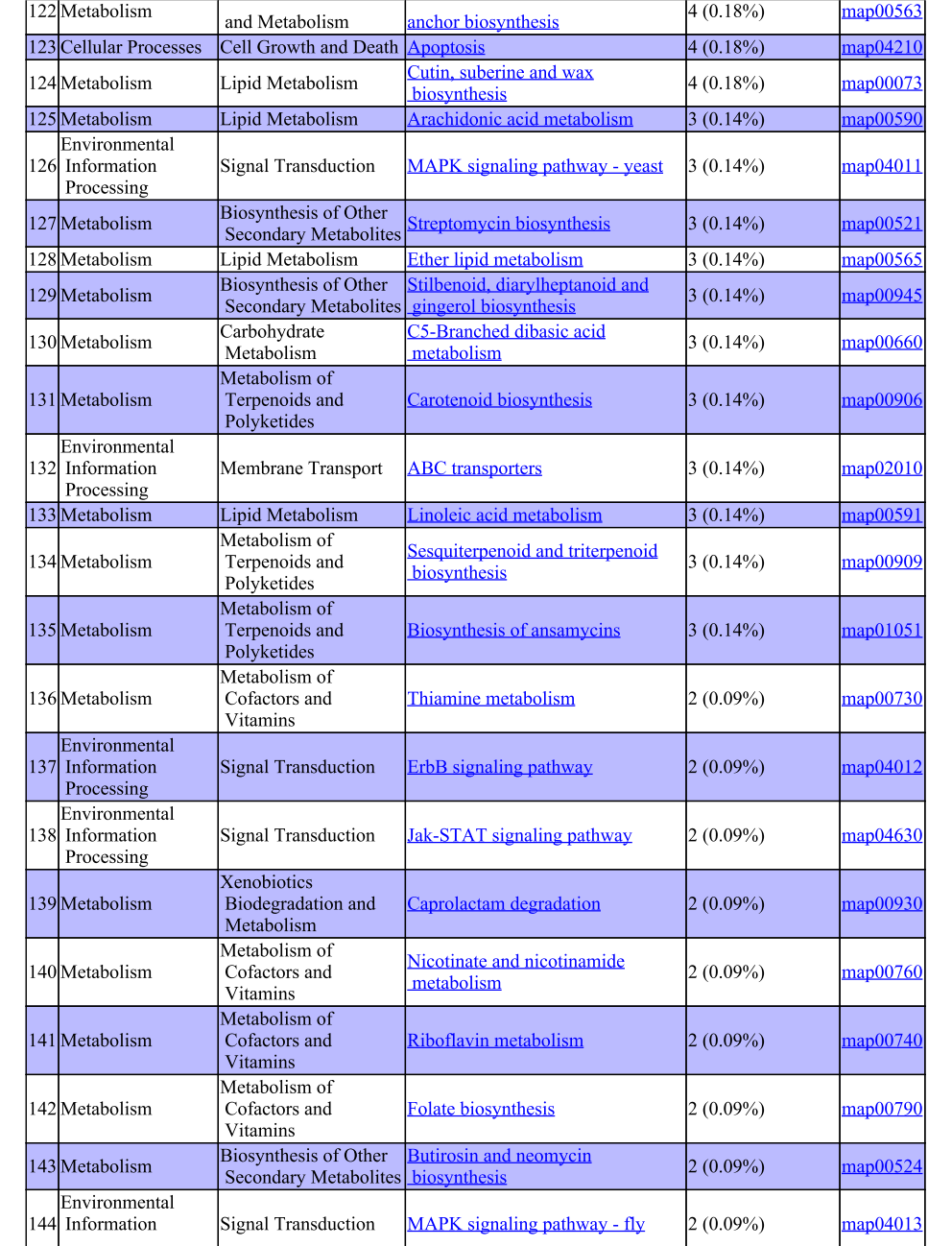


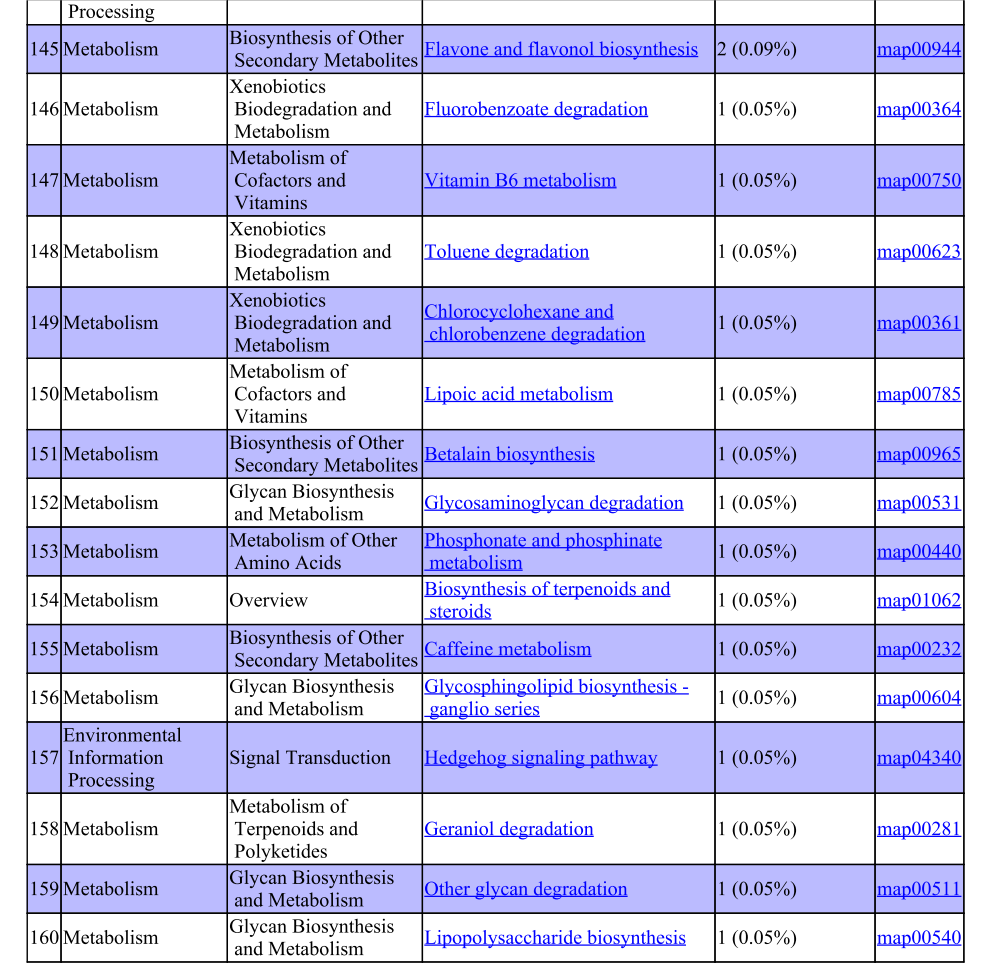


1. Illumina dataset


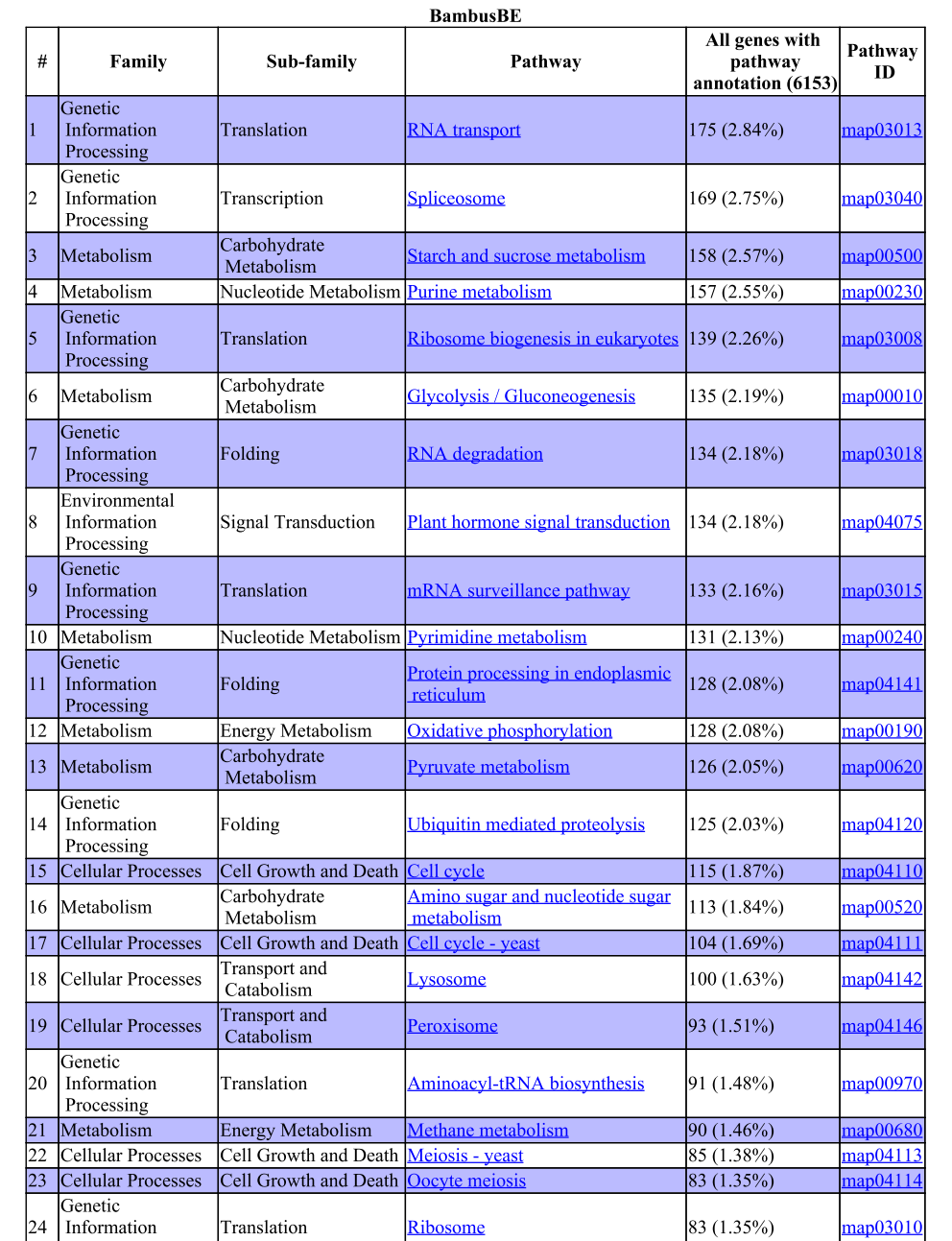


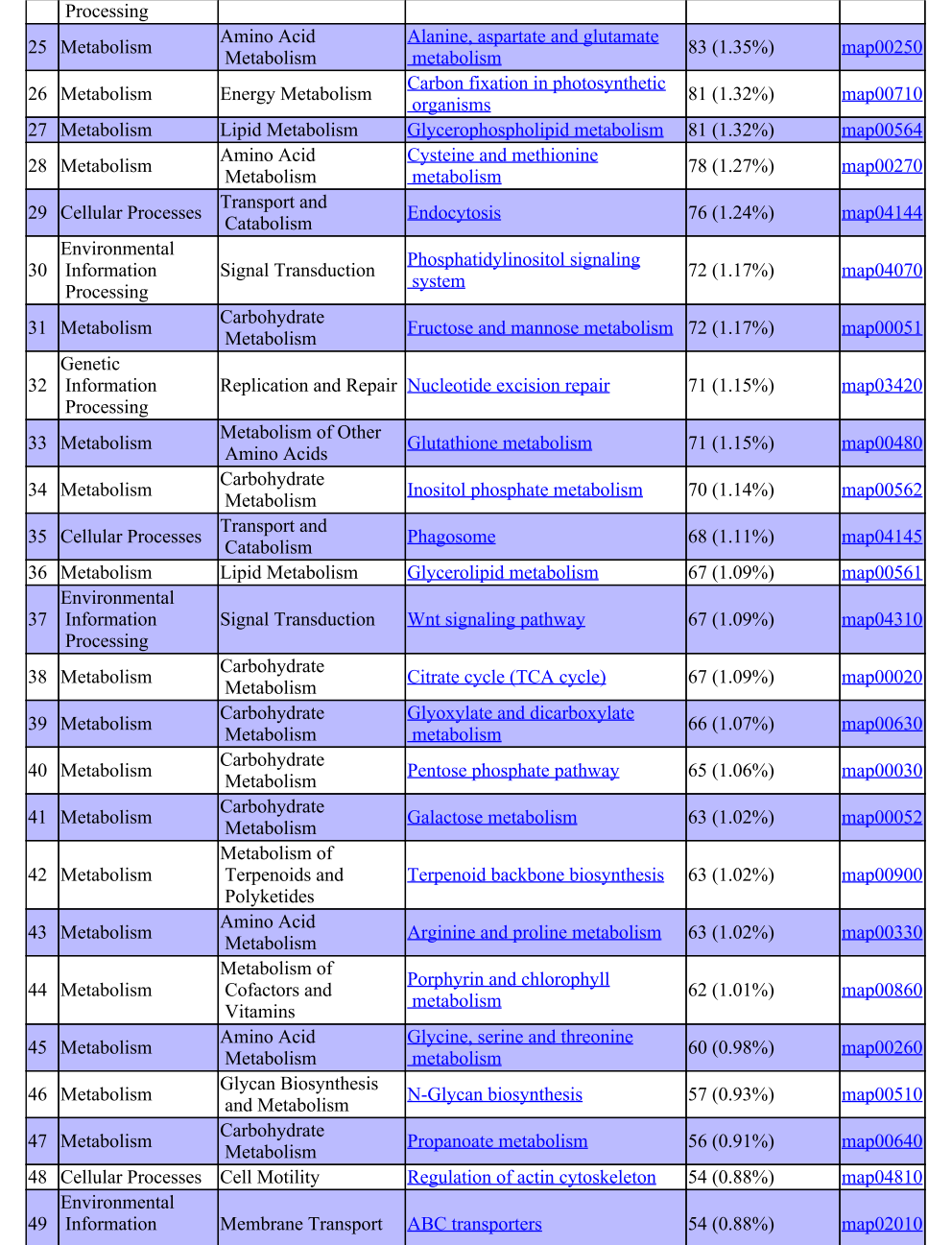


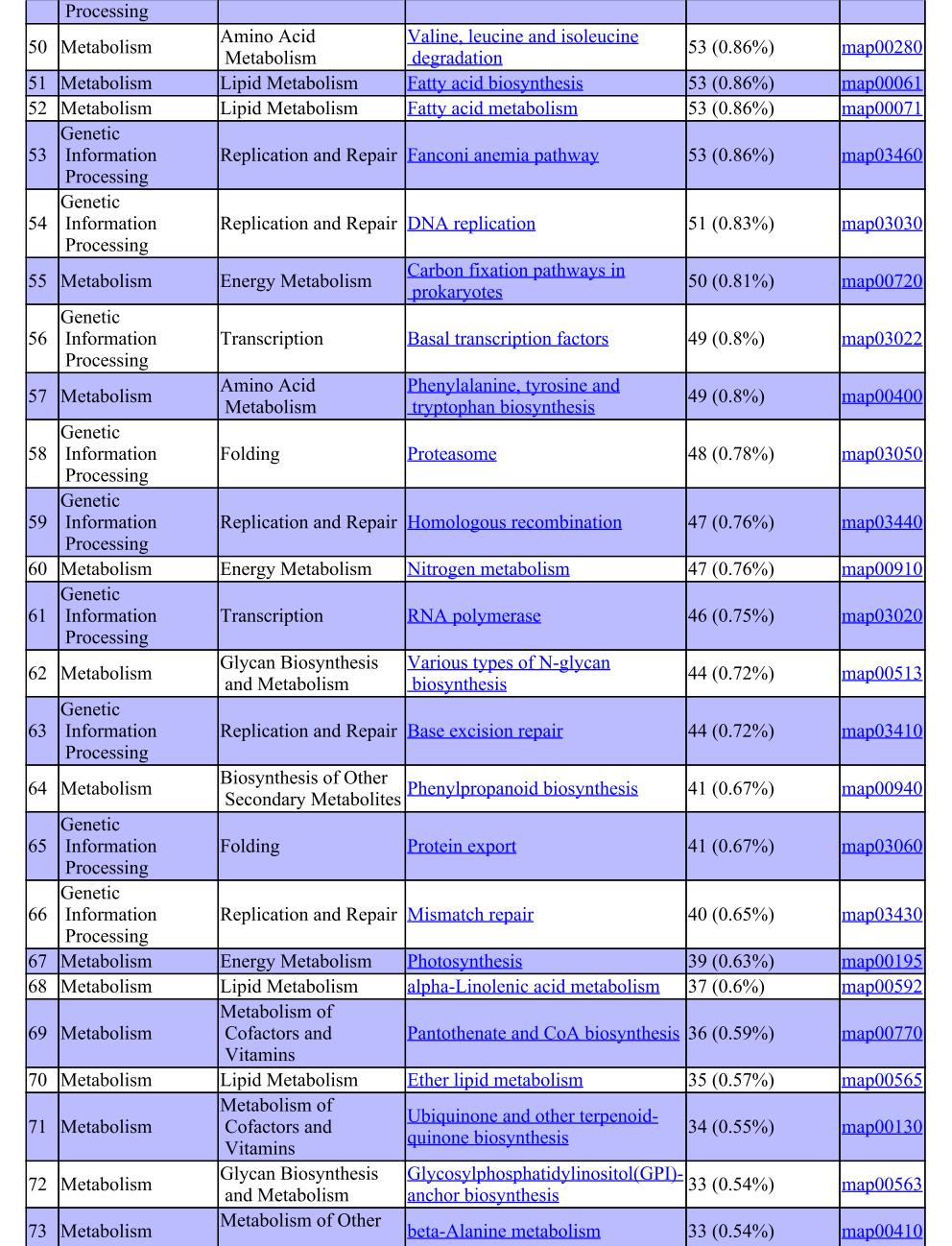


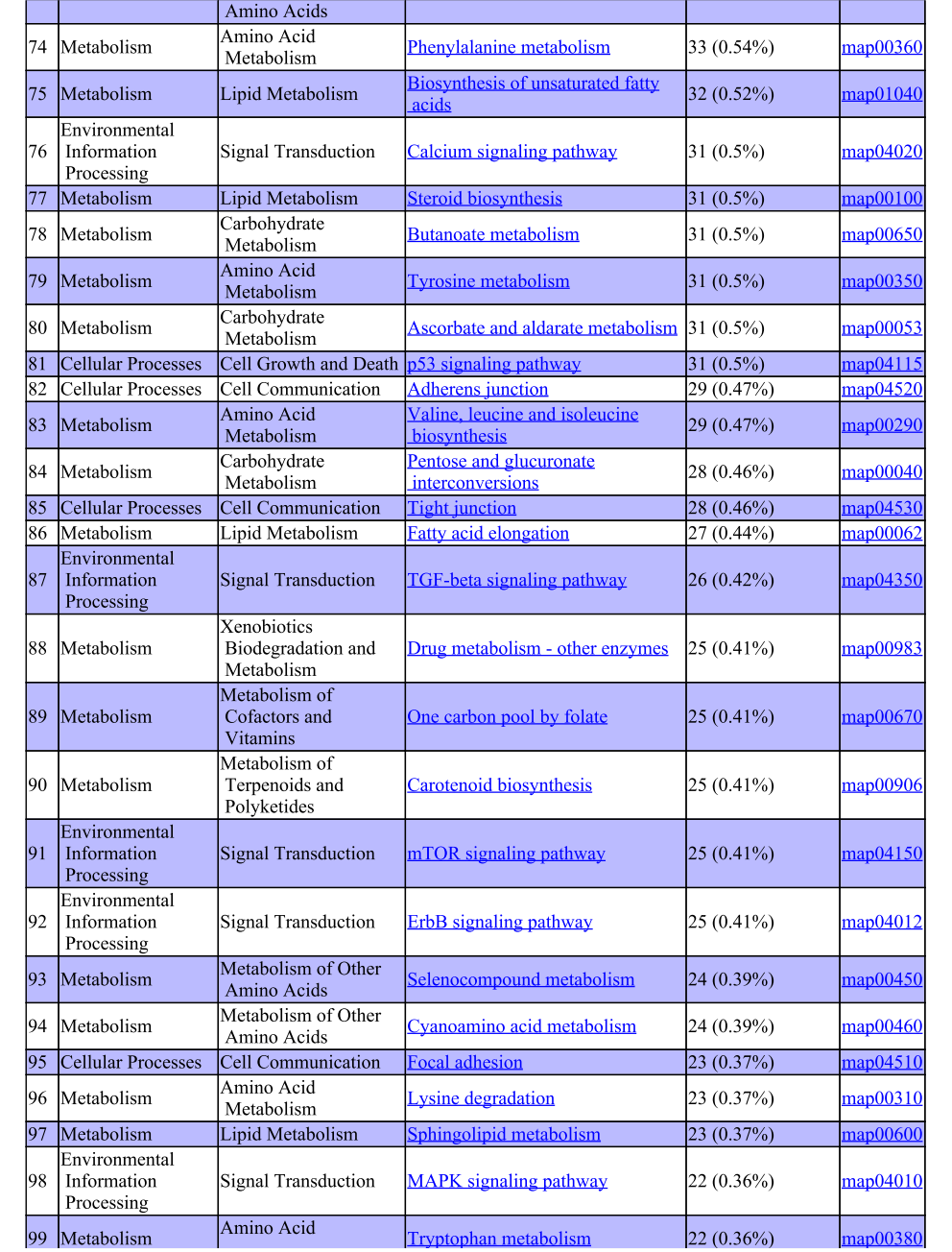


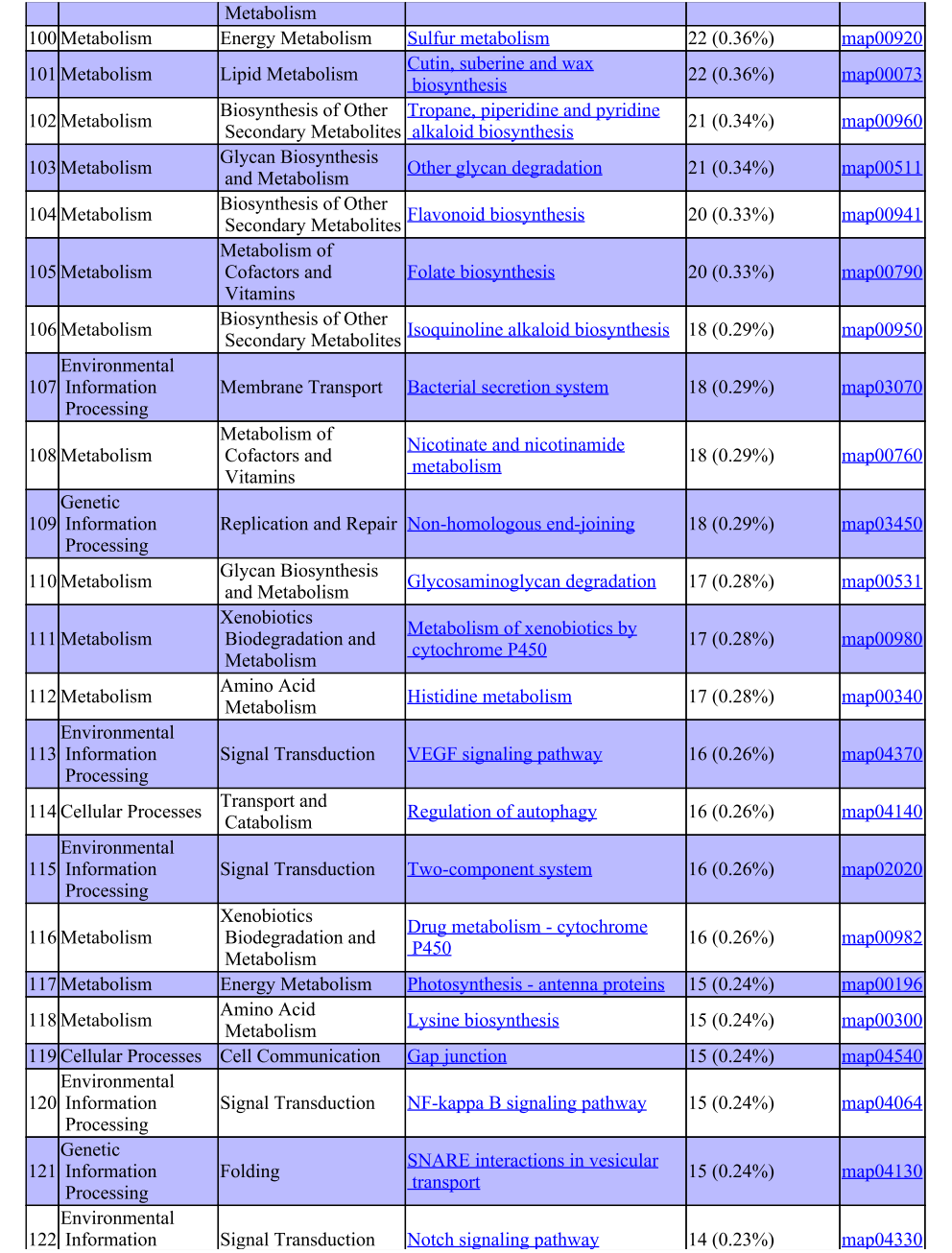


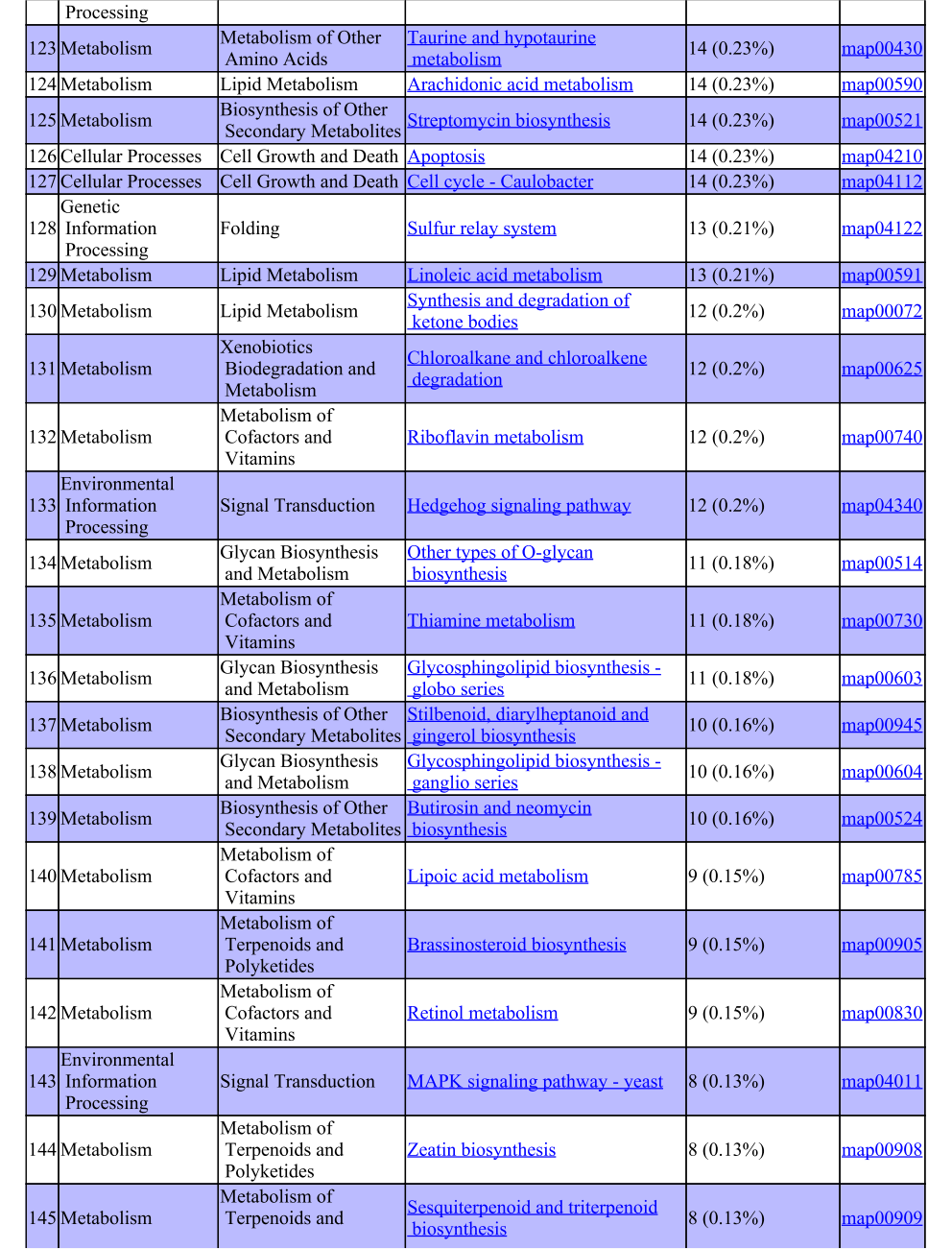


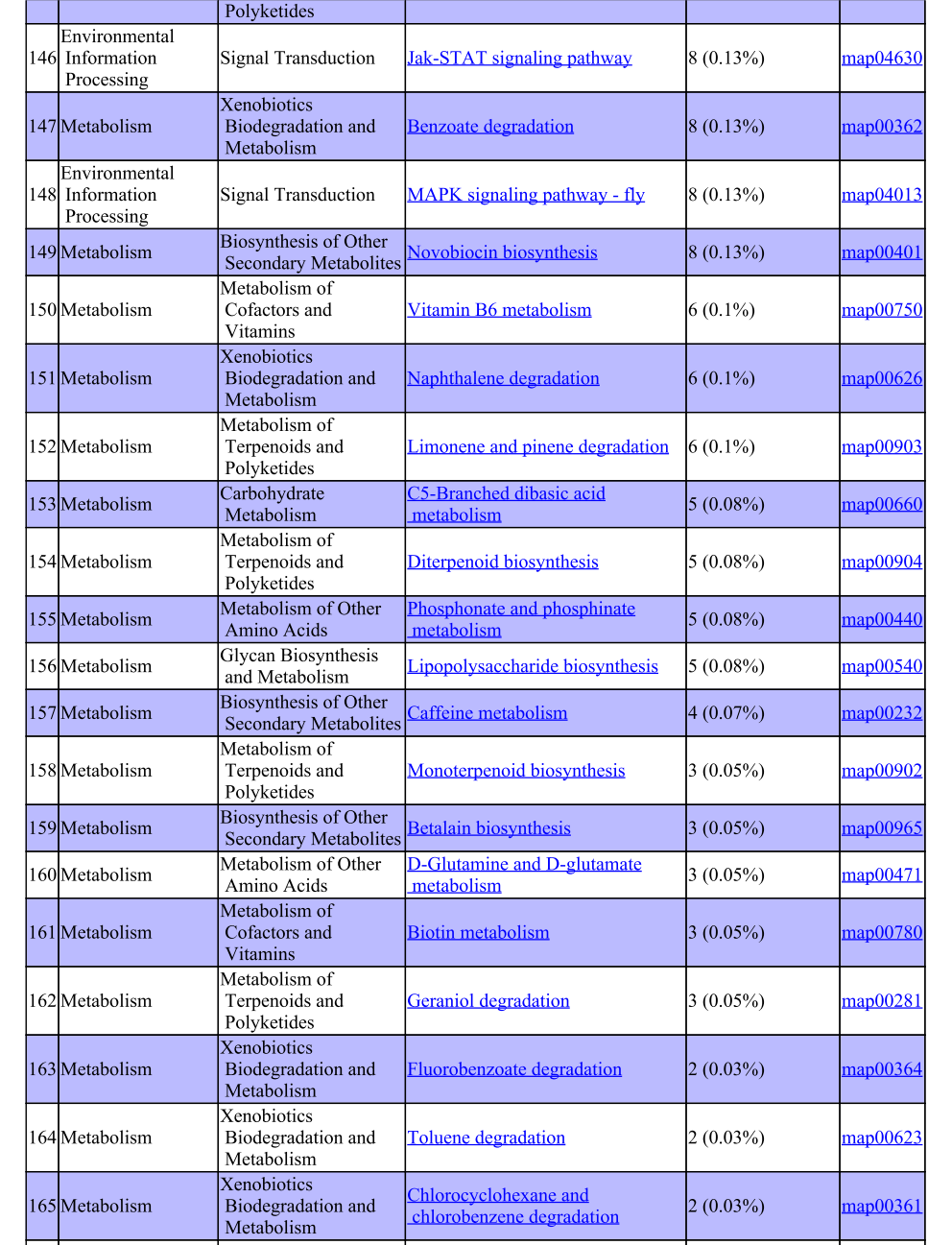


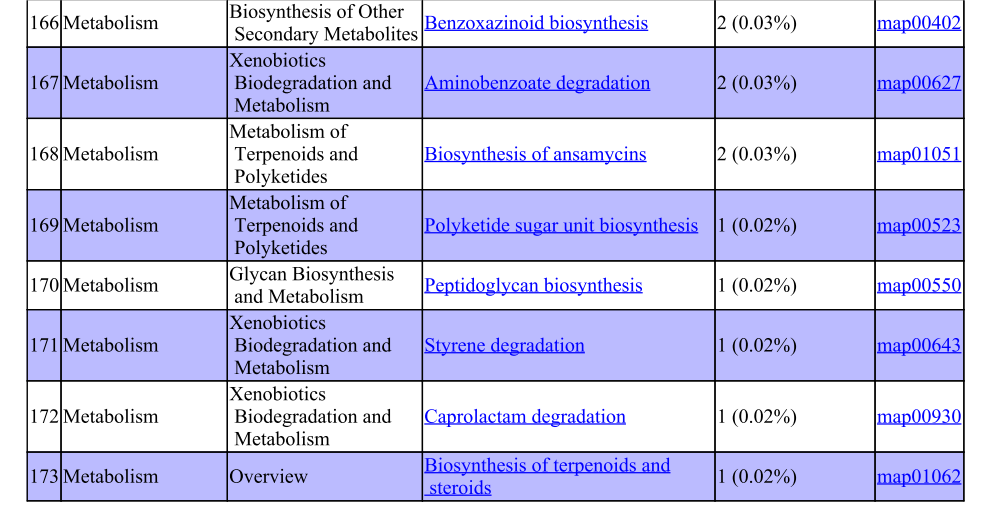


1. Hybrid dataset


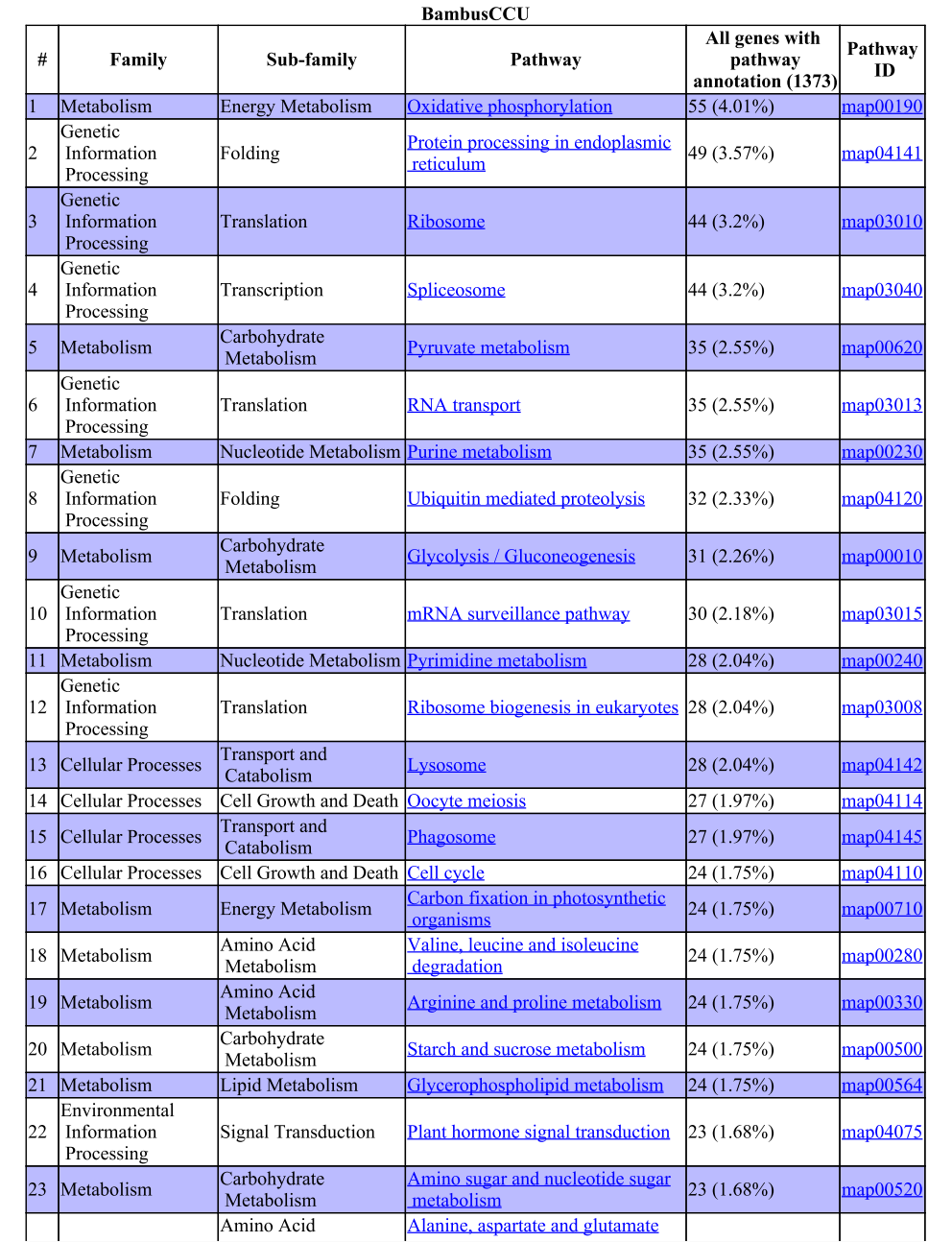


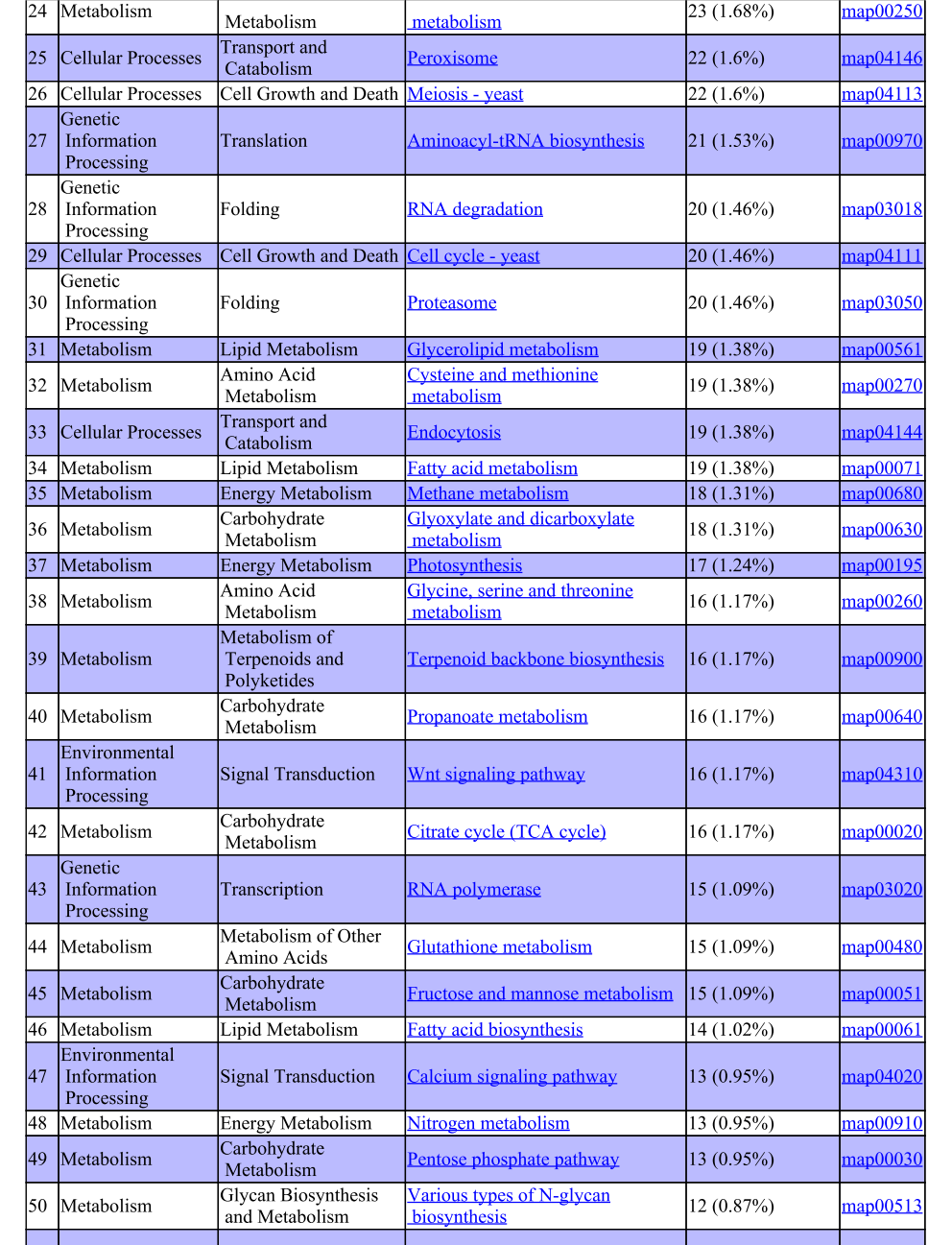


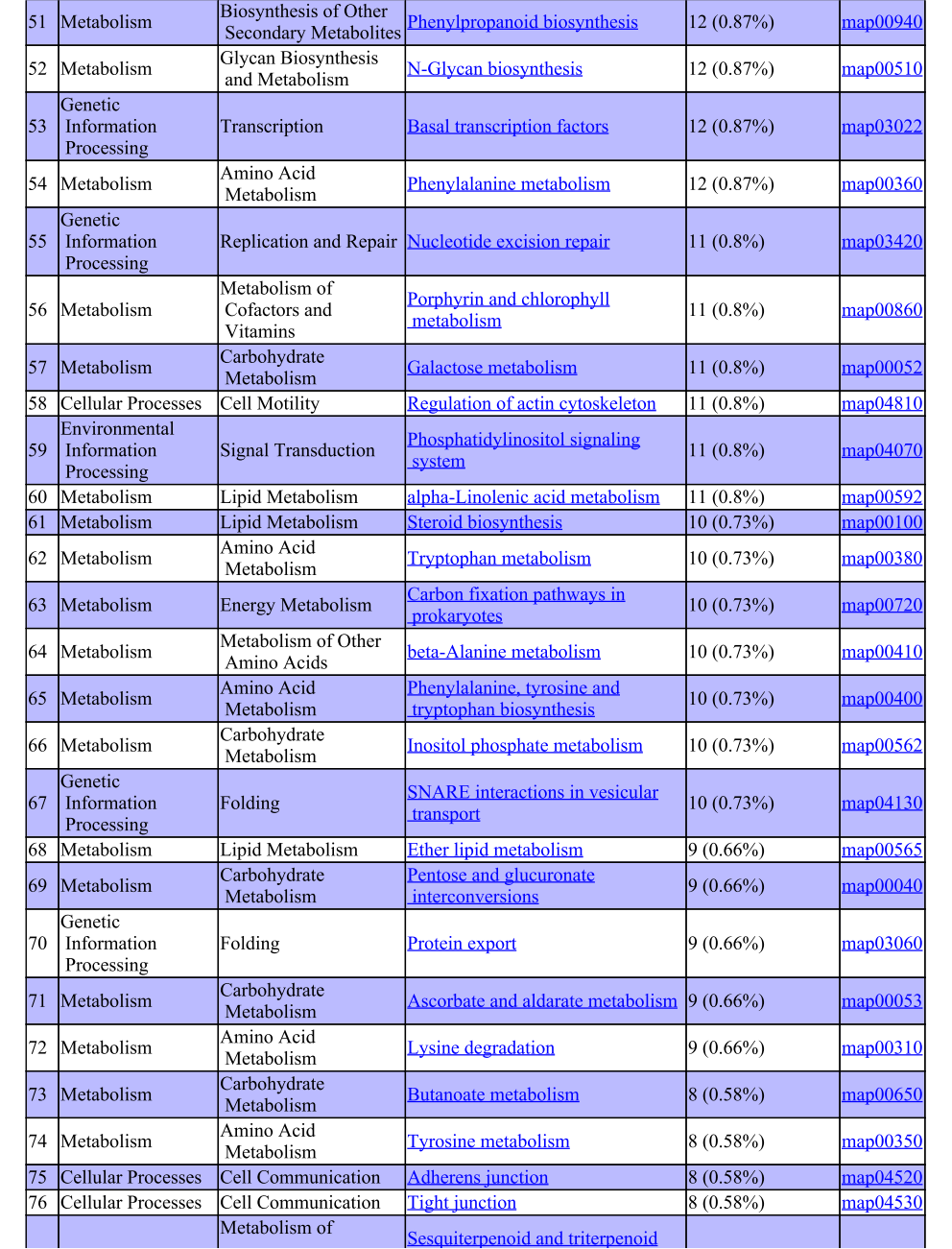


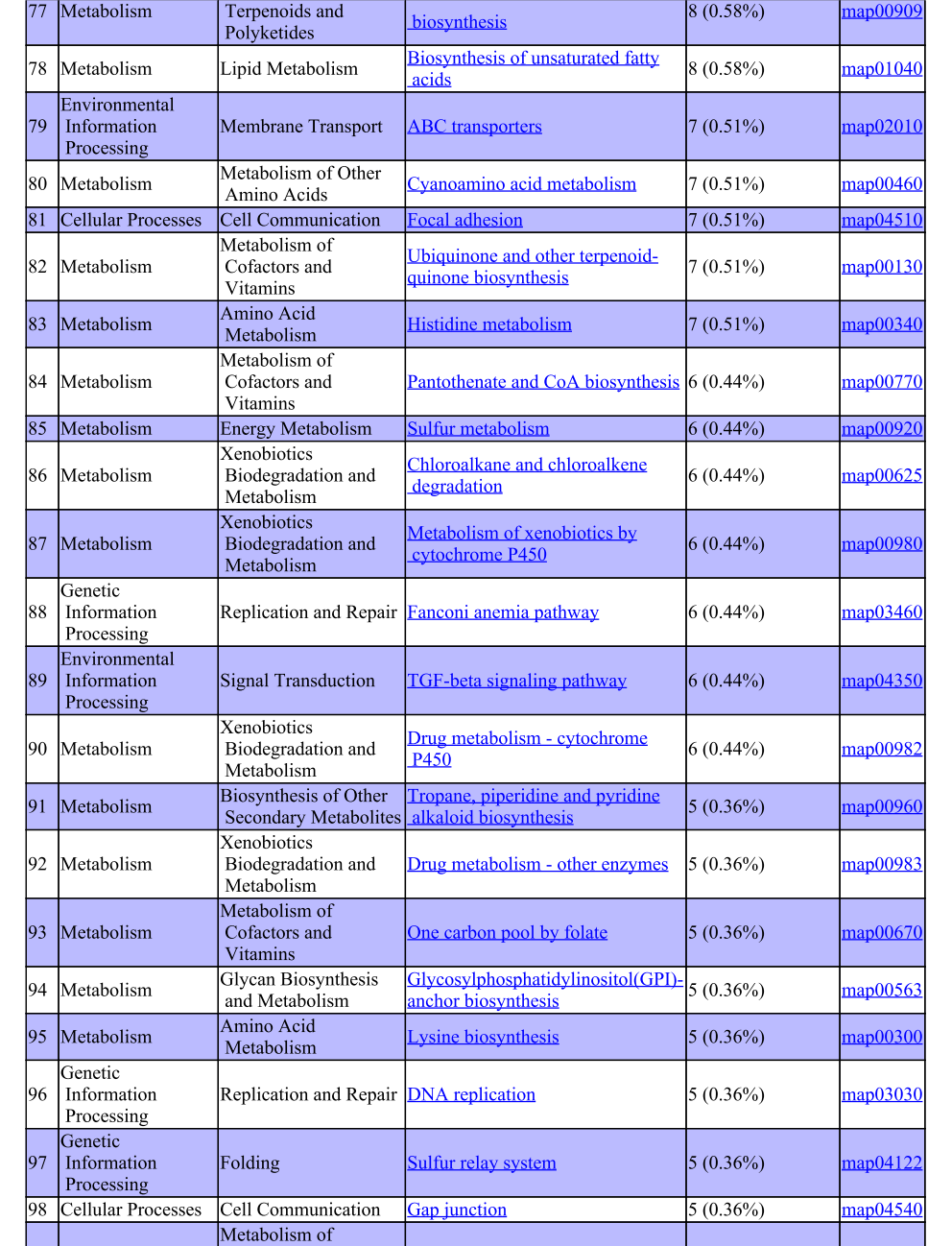


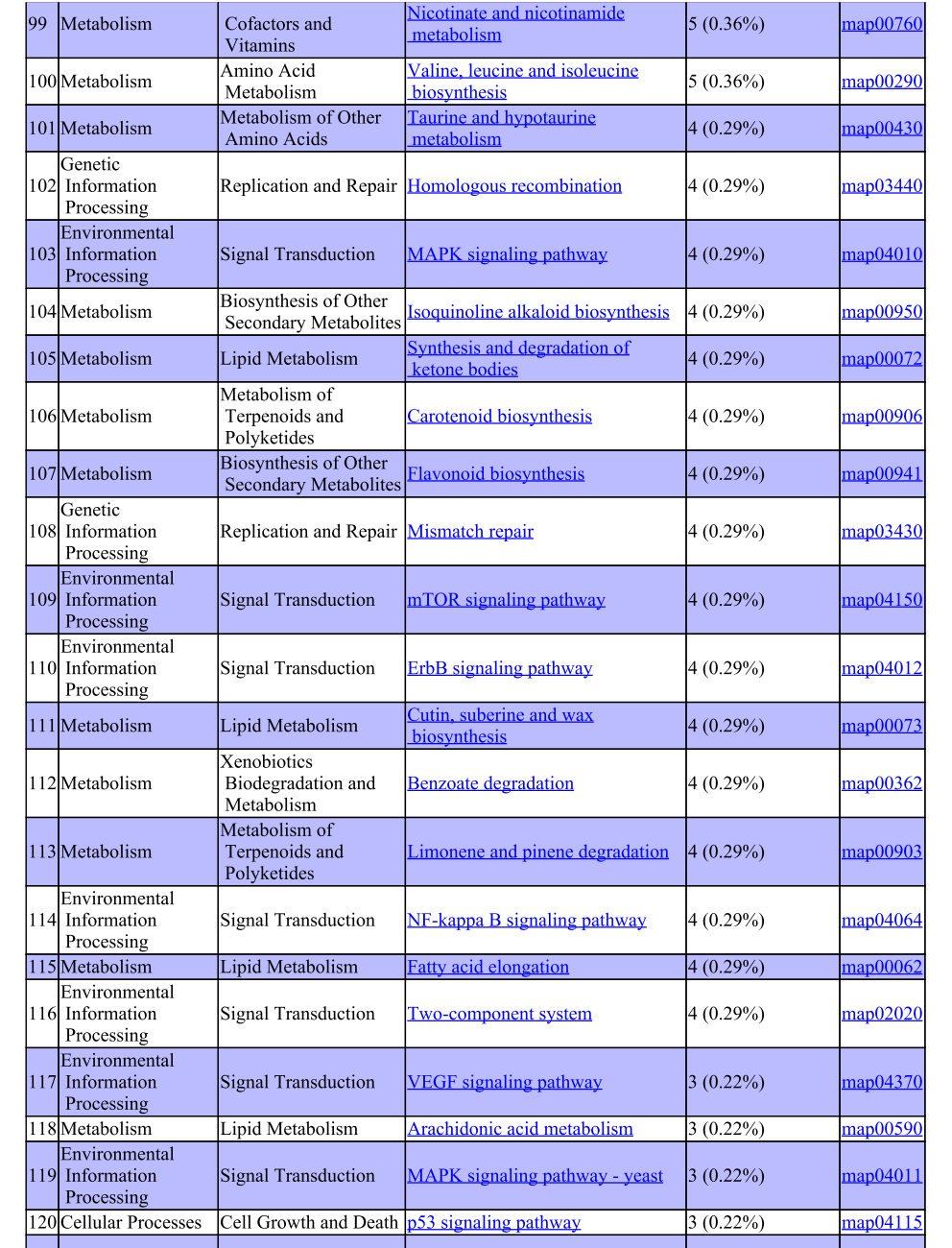


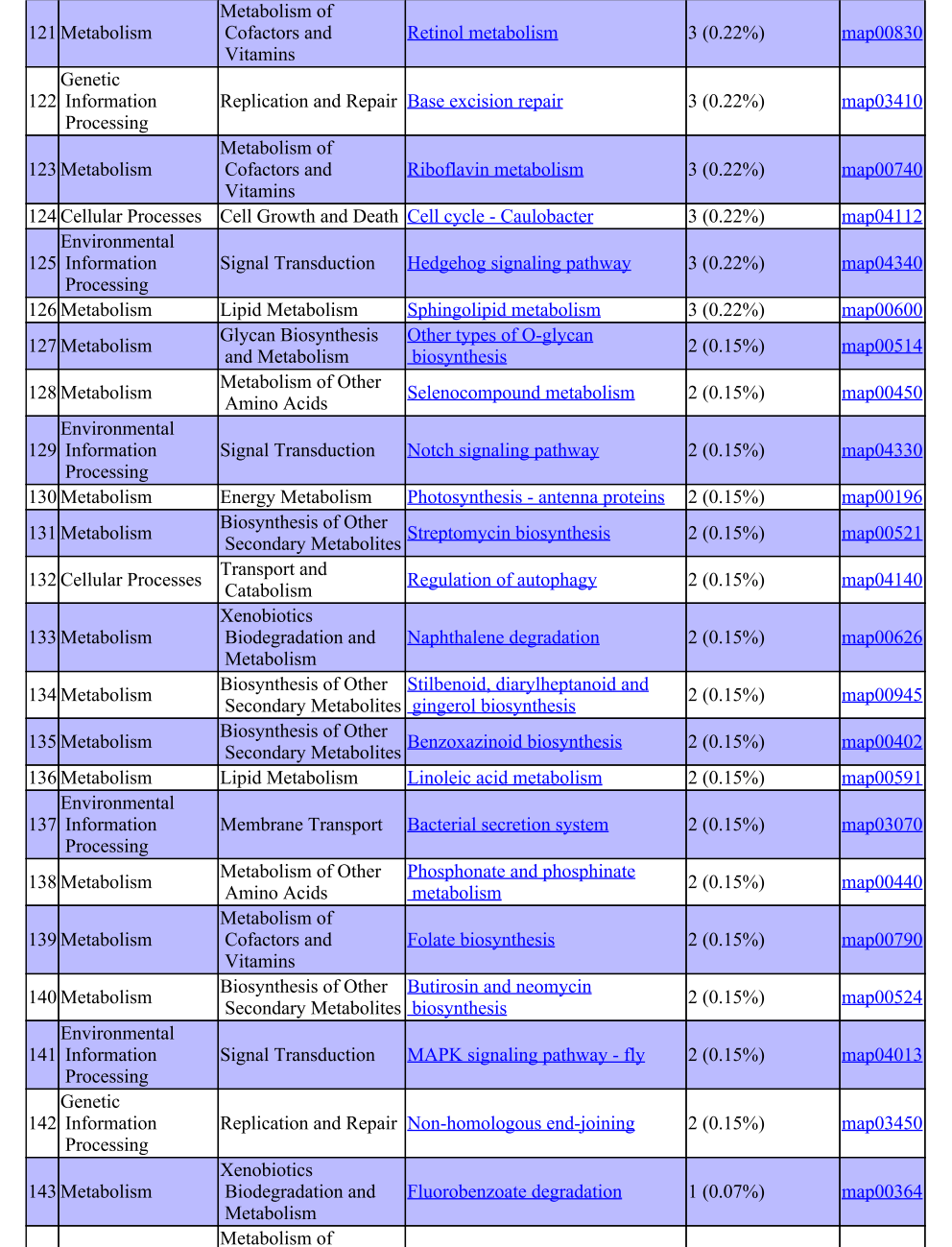


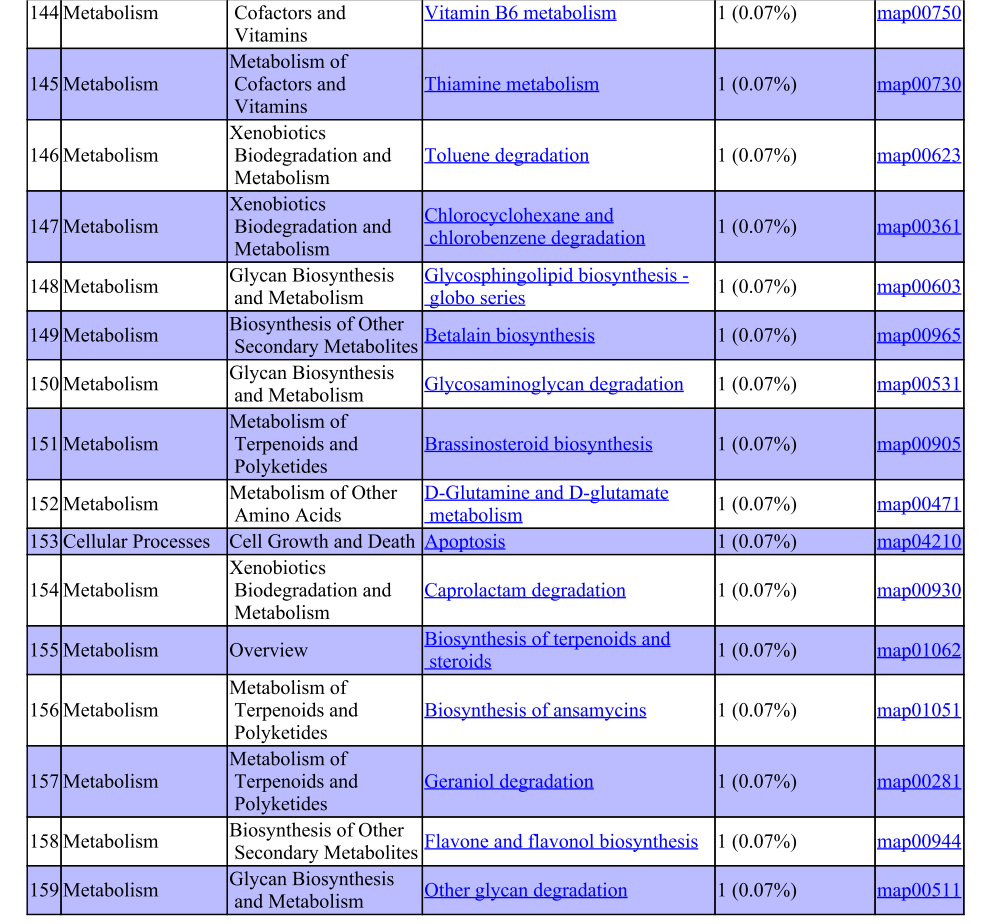

Supplement: Additional file 3 — Unigene metabolic pathway analysis from three B. edulis transcriptome datasets. Unigene metabolic pathway analysis from three B. edulis transcriptome datasets. (A) 454 dataset. (B) Illumina dataset. (C) Hybrid dataset. These sequences were analysis by Kyoto Encyclopedia of Genes and Genomes (KEGG) pathway database. [file 1471-2229-14-179-S3.docx]
