# Supplementary material for: BeMADS1 is a key to delivery MADSs into nucleus in reproductive tissues-De novo characterization of Bambusa edulis transcriptome and study of MADS genes in bamboo floral development
Source: BMC Plant Biol. 2014 Jul 2;14:179. doi: 10.1186/1471-2229-14-179 (PMC4087239; doi:10.1186/1471-2229-14-179)
Supplement: Additional file 4 — Accession numbers of B. edulis floral development-related MADS genes. The accession numbers of B. edulis flower development-related MADS genes as deposited into the NCBI database. [file 1471-2229-14-179-S4.docx]

| Gene name | Accession no. |
| --- | --- |
| BeMADS1 | KJ002710 |
| BeMADS2 | KJ002711 |
| BeMADS3 | KJ002712 |
| BeMADS4 | KJ002713 |
| BeMADS5 | KJ002714 |
| BeMADS6 | KJ002715 |
| BeMADS7 | KJ002716 |
| BeMADS8 | KJ002717 |
| BeMADS13 | KJ002718 |
| BeMADS14 | KJ002719 |
| BeMADS15 | KJ002720 |
| BeMADS16 | KJ002721 |
| BeMADS18 | KJ002722 |
| BeMADS21 | KJ002723 |
| BeMADS34 | KJ002724 |
| BeMADS58 | KJ002725 |
