# Supplementary figures and images for: BeMADS1 is a key to delivery MADSs into nucleus in reproductive tissues-De novo characterization of Bambusa edulis transcriptome and study of MADS genes in bamboo floral development
Source: BMC Plant Biol. 2014 Jul 2;14:179. doi: 10.1186/1471-2229-14-179 (PMC4087239; doi:10.1186/1471-2229-14-179)

## Slide 1
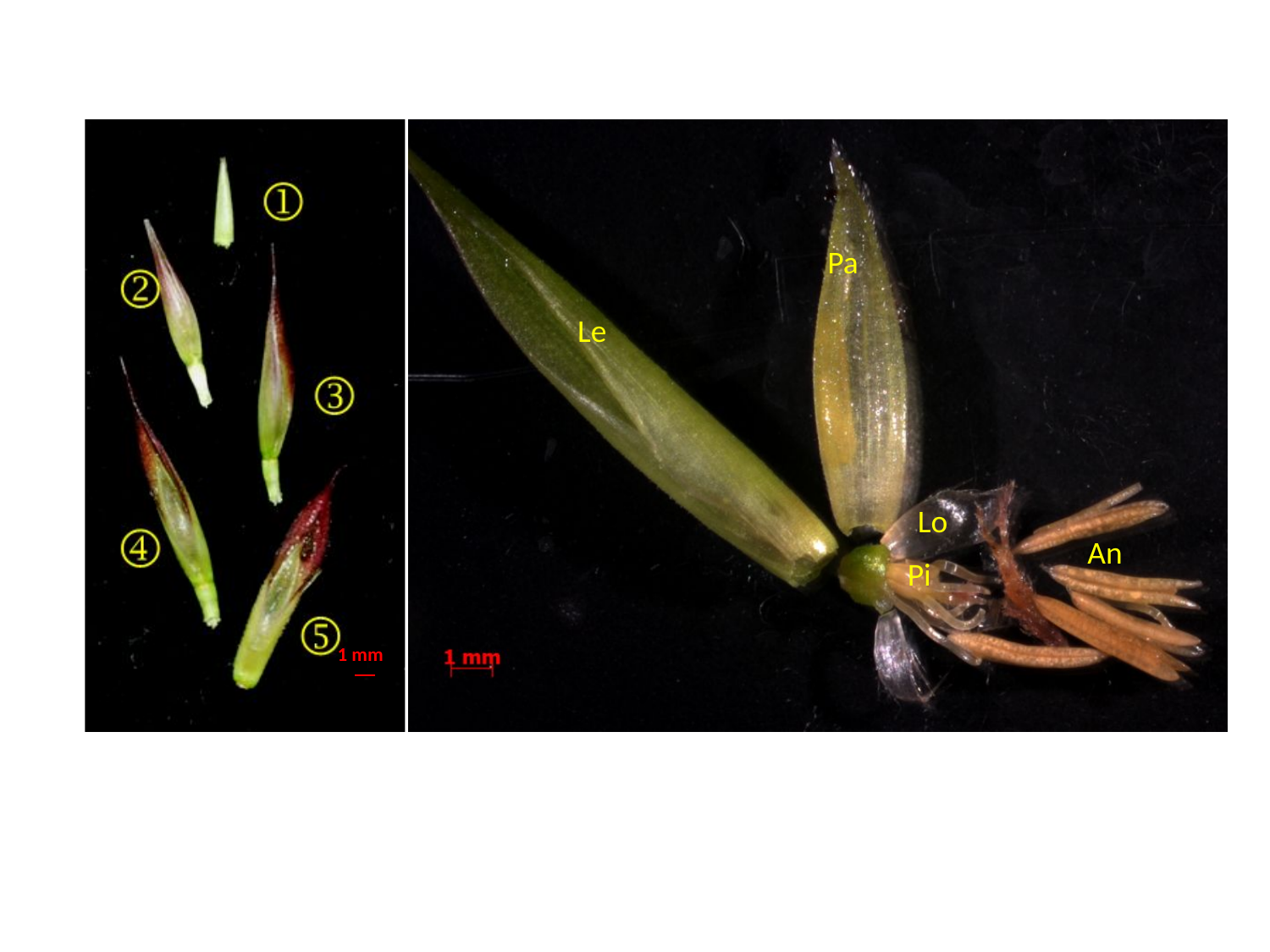

Pa
Le
Lo
An
Pi
1 mm

Supplement: Additional file 5 — Flower material for qRT-PCR. The flower material for qRT-PCR. (Left) Each spikelet in B. edulis has multiple florets. The florets were numbered 1–5, young to old. Bar = 1 mm. (Right). The mature florets are enclosed by two bracts called the palaea (Pa) and lemma (Le). The perianth of each floret is represented by two transparent scales called lodicules (Lo). There are generally three anthers (An) and a pistil (Pi) with two hairy stigmatic lobes. Bar = 1 mm. [file 1471-2229-14-179-S5.pptx]

## Slide 1
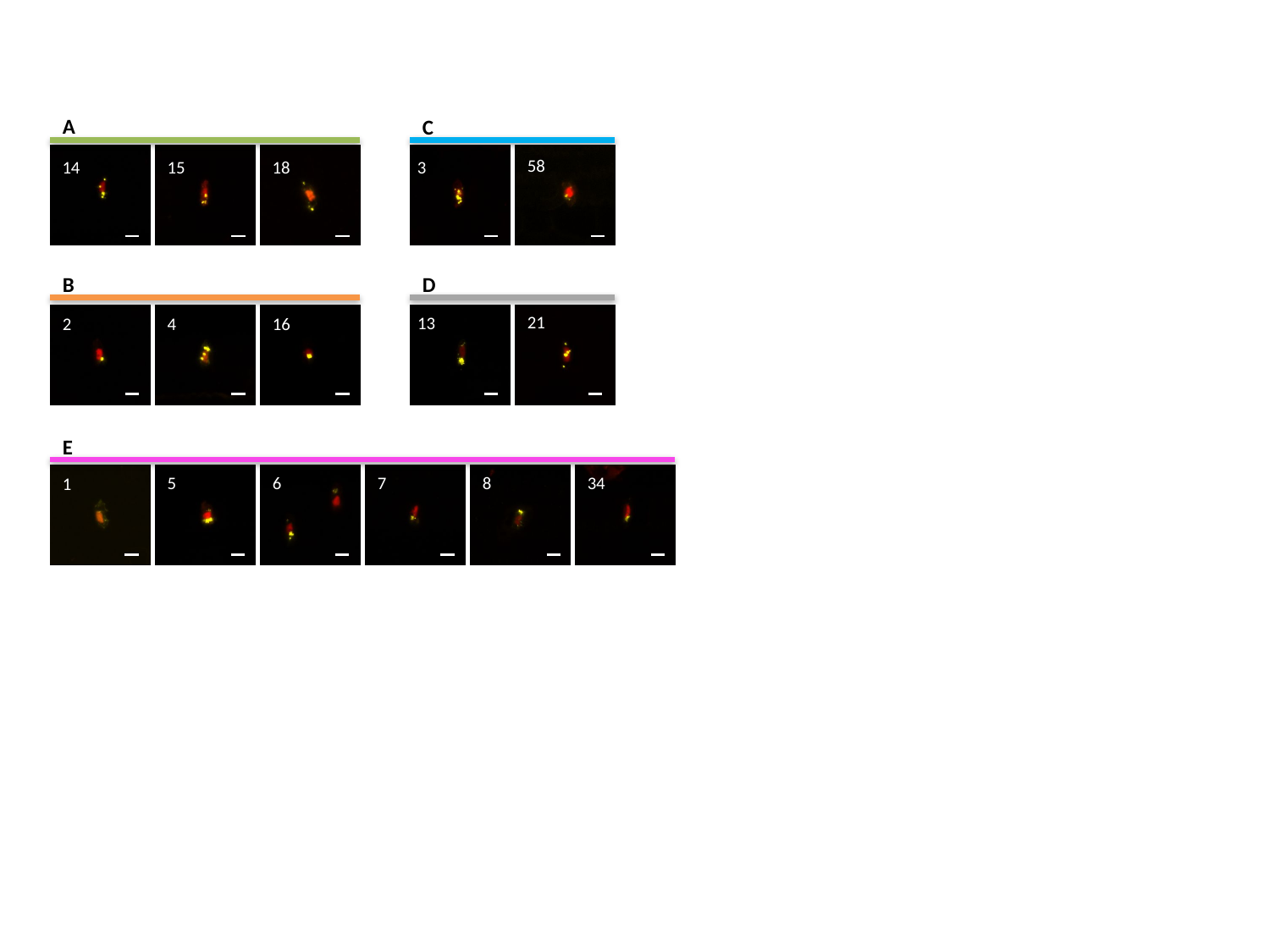

A
C
58
3
14
15
18
B
D
21
13
2
4
16
E
5
6
7
8
34
1

Supplement: Additional file 7 — Subcellular localization of BeMADS fused with fluorescent proteins in B. edulis leaves. Subcellular localization of BeMADS fused with fluorescent proteins in B. edulis leaves. Plasmids harboring a YFP fusion with different BeMADS proteins (Yellow signals, the number indicates the gene name) driven by the 35S promoter were transiently expressed in B. edulis leaves. The functional classification according to A/B/C/D/E class are indicated at the top of the panels. These plasmids were delivered by particle bombardment. The NLS domain of VirD2 fused with mCherry was used as the nuclear marker (in red color). Only the merged images are shown. Bar = 20 μm. [file 1471-2229-14-179-S7.pptx]
