# Supplementary material for: BeMADS1 is a key to delivery MADSs into nucleus in reproductive tissues-De novo characterization of Bambusa edulis transcriptome and study of MADS genes in bamboo floral development
Source: BMC Plant Biol. 2014 Jul 2;14:179. doi: 10.1186/1471-2229-14-179 (PMC4087239; doi:10.1186/1471-2229-14-179)
Supplement: Additional file 6 — Primer list. The primer list in this study. Primers for full length genes were used in PCR with DNA from the Bambusa edulis BAC library. [file 1471-2229-14-179-S6.docx]

The Primers for q-PCR

| BeMADS1-qPCR_F | GCATATCCAGTGTGTCCATTAGG |
| --- | --- |
| BeMADS1-qPCR_R | TGCACTACATATTTCCTGGCAAG |
| BeMADS2-qPCR_F | CGACTACTGCTCCCTCCAAG |
| BeMADS2-qPCR_R | GATCGATCTCCGCACTAAGG |
| BeMADS3-qPCR_F | CGATCACATGGTTCCGTATGATT |
| BeMADS3-qPCR_R | GATTTCACCTAACGCGTCCAATA |
| BeMADS4-qPCR_F | ATCTTCTCATTCCTCCTGTGCAT |
| BeMADS4-qPCR_R | GGGTCAAGAAGGAGAACGATAAC |
| BeMADS5-qPCR_F | ACATCTTGCAGTTGTTGTTCCTT |
| BeMADS5-qPCR_R | ATATTCTTGGTGAGGACTTGGGT |
| BeMADS6-qPCR_F | AAACAGCTAGAATGTGCTCTGTC |
| BeMADS6-qPCR_R | TGGTTCTATAGTTGCTGCTACCT |
| BeMADS7-qPCR_F | AAAGGAATTTGCTCGGTGAAGAT |
| BeMADS7-qPCR_R | ACATTTGTACCCTTCTCTGGAGT |
| BeMADS8-qPCR_F | AGTATCTCAAACTGAAAGCACGG |
| BeMADS8-qPCR_R | ATCAACCATATGCTGTGTCCTTG |
| BeMADS13-qPCR_F | CTGGCTGCAGAGATCAATTTCAT |
| BeMADS13-qPCR_R | AAGCACTTCATGTCCAGCAC |
| BeMADS14-qPCR_F | GCGAAGGTTGAGACAATACAGAA |
| BeMADS14-qPCR_R | CTCCTCTTTTGAAGCTCGGAAAT |
| BeMADS15-qPCR_F | CTGAATCTGAAAGCGAGGGAAAT |
| BeMADS15-qPCR_R | GATGTGCTTCAATGAACTCTCCA |
| BeMADS18-qPCR_F | CTCCAACAATTGGAACAACAA |
| BeMADS18-qPCR_R | GACTTCTCCTTCTTCTGAAGCTC |
| BeMADS21-qPCR-F | AGATCAAGAGAATCGAGAACACG |
| BeMADS21-qPCR_R | GGCCTTCTTGTACCTCTCAATTG |
| BeMADS34-qPCR_F | ACTAACTCTTGTGTGCATGACTG |
| BeMADS34-qPCR_R | AGCTGCCTCTACCTCGATCT |
| BeMADS58-qPCR_F | AATCGAGTACATGCAAAGAAGGG |
| BeMADS58-qPCR_R | AGTTTGTTGGATCGTAATGGACC |

The primers for BeMADS full length sequences

| BeMADS1_full_length_F | ATGGGTCGCGGGAAGGTGGA |
| --- | --- |
| BeMADS1_full_length_R | CATCCAACCTGCATATCCAG |
| BeMADS2_full_length_F | ATGGGGCGCGGGAAGATCGA |
| BeMADS2_full_length_R | GTTGTTCTCCTGCAGGTTGG |
| BeMADS3_full_length_F | ATGGGGAGGGGAAAGATTGA |
| BeMADS3_full_length_R | ACGCGTCCAATAAGGAGAGT |
| BeMADS4_full_length_F | ATGGGGCGCGGCAAGATCGA |
| BeMADS4_full_length_R | CTTGTCTTCCTGCAGGTTGG |
| BeMADS5_full_length_F | ATGGGCCGCGGGAAGGTTGA |
| BeMADS5_full_length_R | TTCATTGTTCAAGTGGTCCA |
| BeMADS6_full_length_F | ATGGGTAGGGGGAACGTGGA |
| BeMADS6_full_length_R | AAGAACCCACCCCAGCATGA |
| BeMADS7_full_length_F | ATGGGGAGGGGGCGGGTGGA |
| BeMADS7_full_length_R | GAGTAGCCACGGGGTCATGT |
| BeMADS8_full_length_F | ATGGGGAGGGGTAAGGTGGA |
| BeMADS8_full_length_R | AGGTAGCCATGTAGGCATGA |
| BeMADS13_full_length_F | ATGGGGAGGGGAAGAATTGA |
| BeMADS13_full_length_R | GAACTGAGGAGCTGCCTCGG |
| BeMADS14_full_length_F | ATGGGGCGCGGGAAGGTGCA |
| BeMADS14_full_length_R | GCCGTCGATGTGGCTCACCA |
| BeMADS15_full_length_F | ATGGGGCGCGGCAAGGTGCA |
| BeMADS15_full_length_R | AGCATTGAGATGGCTCAGCA |
| BeMADS16_full_length_F | ATGGGGCGCGGCAAGATCGA |
| BeMADS16_full_length_R | ACCGAGGCGCATGTCGTGGG |
| BeMADS18_full_length_F | ATGGGGCGCGGGCCGGTGCAGCTAC |
| BeMADS18_full_length_R | TCATGTGTTGTTATTGACACAGCGT |
| BeMADS21_full_length_F | ATGGGGAGGGGGAAGATTGA |
| BeMADS21_full_length_R | GAGGAAGCCTTTGCCGGCGG |
| BeMADS34_full_length_F | ATGGGTCGCGGCAAGGTGGT |
| BeMADS34_full_length_R | CGCTGGCGGCAGAGACGGGT |
| BeMADS58_full_length_F | ATGGGGAGGGGAAAGATTGA |
| BeMADS58_full_length_R | CCTTTCATCTGAGTCGAAAG |
